# Putting mental health deinstitutionalisation back on track: a scoping review of what empirically hinders and drives deinstitutionalisation of adults who experience mental illness

**DOI:** 10.1186/s12889-025-24496-0

**Published:** 2025-11-26

**Authors:** Luís Sá-Fernandes, Beatrice Sacchetto, Johann Pires, José H. Ornelas, Maria João Vargas-Moniz

**Affiliations:** 1 ISPA – Instituto Universitário, Rua Jardim do Tabaco, nº34, Lisbon, 1140-041 Portugal; 2 Applied Psychology Research Centre Capabilities and Inclusion, Rua Jardim do Tabaco, nº34, Lisbon, 1140-041 Portugal

**Keywords:** Deinstitutionalisation, Mental health, Scoping review, Community inclusion

## Abstract

**Background:**

Mental health deinstitutionalisation continues to be a global human rights priority. After over half a century, the discharge to the community often means the transition to smaller-scale institutions, segregation environments, and limited opportunities for community inclusion. This scoping review aims to identify what hinders and drives the deinstitutionalisation process of adults experiencing mental health challenges.

**Method:**

A scoping review was conducted following the Joanna Briggs Institute methodology and reported under the PRISMA extension for scoping reviews (PRISMA-ScR). A systematic search of four electronic databases, PubMed, APA PsycINFO, Web of Science, and Scopus, was undertaken between January and March 2024. Only empirical studies focusing on the deinstitutionalisation process of adults with mental health challenges, published in English, from 1991 to 2024 were eligible for inclusion. A template in Microsoft Excel was created for data extraction. Results were descriptively synthesised and organised into the system change framework’s four fundamental dimensions (norms, resources, regulations, and operations).

**Results:**

A total of 57 studies were included. Most of those included studies, 53% were qualitative (*n* = 30), 60% were published from 2014 to 2024 (*n* = 34), 26% were from North Americas (*n* = 15), and 25% were from Europe (*n* = 14). Factors that hindered mental health deinstitutionalisation included the exclusiveness of the medical model, social discrimination, insufficient community services, transinstitutionalisation, lack of support for community inclusion, most funds allocated to institutionalisation, economic incentives for institutionalisation, institutional policies, inefficient governance, professional control, and limited advocacy. Drivers included a model for community inclusion, an inclusive society, resourcing community alternatives, independent housing, individualised context-oriented support, economic pressures, policy and legal reform, consumer participation in services, and consumer advocacy.

**Conclusions:**

The study findings constitute an important basis to inform the ongoing or future deinstitutionalisation processes of adults with mental illness diagnoses.

**Supplementary information:**

The online version contains supplementary material available at 10.1186/s12889-025-24496-0.

## Background

Mental health deinstitutionalisation continues to be a global priority in terms of human rights regulation and legislation [1, 2]. Despite legislative and policy key contributions towards deinstitutionalisation, namely the English Mental Health Act in 1959 [3], the Community Mental Health Centres Act of 1963 [4], or the Basaglia’s 180 Law in 1978 [5], the movement from mental health institutions towards the community often means the transition to smaller-scale institutions, where segregation and social exclusion tend to persist and prevail [6, 7]. At the same time, the survivor’s movement continuously advocates for the release of the people who are still in large-scale wards, calling for the end of institutionalisation [8–11].

Deinstitutionalisation is defined as the transition from institutionalised confinement to the community, requiring the development of policies, community support services, or alternative user-led services that guarantee full rights and citizenship for people with psychiatric disabilities [12–17]. In contrast, institutionalisation means segregation from society, unconsented treatments, trials, and powerlessness regarding abuses, humiliation, or control over one’s life, all constituting major violations of basic human rights [18–22].

The deinstitutionalisation movement has been largely influenced since the 1950 s and 1960 s by humanitarian, economic, and social factors [23–25]. Deinstitutionalisation became a relevant policy arena due to activists, journalists, and social scientists (e.g., Beers [26]; Deutsch [27]; Goffman [28]) unveiling the negative effects of institutionalisation, along with the civil rights movements, and the development of cost-effective alternatives to large institutions [29]. The deinstitutionalisation processes still raise long-standing disputes and arguments about whether closing psychiatric hospitals leads to increases in prison populations [25, 30, 31] or what model of care better guides deinstitutionalisation and mental health practices (e.g., Szasz [32]; Wolf [33]; Albee and Joffe [34]; Minkowitz [22]). Deinstitutionalisation presents a complex and long-standing paradox. According to Bachrach [35], it involves not only releasing people from institutions but also embracing a philosophy that values individual rights and social change. However, simply moving care out of hospitals does not guarantee more humane treatment. Gruenberg [36] highlighted how institutionalisation itself damages confined people by lowering expectations and diminishing their motivation and skills, creating a cycle that deinstitutionalisation aims to break. Estroff [37] points out that most efforts have focused on changing people instead of addressing the social and cultural reasons behind institutionalisation. Grob [4] describes this as the “deinstitutionalisation paradox”, meaning that although people leave psychiatric hospitals, they often face social exclusion, isolation, or even homelessness. Thus, while deinstitutionalisation promises freedom and dignity, it can also lead to new forms of confinement and neglect, creating a difficult tension between protecting rights and providing effective support.

Mental health deinstitutionalisation has induced system reforms in several countries; however, few community services have been implemented, fostering the continued dominance of psychiatric institutionalisation [38–43]. Results of contemporary cross-national studies reveal a modest global evolution of deinstitutionalisation, since the reduction in inpatient beds hasn´t reached half of 1% per year since 2001, and only under half of the world’s nations have adopted deinstitutionalisation policies, which are mostly Western countries [44]; still, in the 27 European Union countries, approximately 1.5 million people continue to live institutionalised lives [45, 46]; contribution for some studies to question whether deinstitutionalisation ever happened due to the continuing movement of re-institutionalisation in other congregated settings [47]. In the past, the failure of deinstitutionalisation in the United States was attributed to the discharge of people from psychiatric hospitals without corresponding funding or the development of community-based services to support their reintegration [48, 49]. This resulted in the creation of large areas of discharged people in deteriorating neighbourhoods, an increase in people placements in nursing homes, contributing to their often-deplorable conditions, and the emergence of phenomena such as the “revolving door” cycle of repeated hospitalisations [49]. Lately, critiques to mental health deinstitutionalisation include arguments such as an unclear or misleading intervention focus, the lack of consensus among stakeholders on organisation and procedures, mental health funding incentives favouring a high bed occupancy rate, the absence of representation of people who experience(d) mental illness in the definition of policies and services, as well as, the inability to grant equitable access to adequate evidence-based community solutions [43, 50–54]. Conversely, Italy’s application of Law 180 in 1978, particularly in regions of Trieste and South Verona, stands as an example of successful deinstitutionalisation, demonstrating that community-based mental health care can effectively replace long-stay hospitalisation without compromising discharged people outcomes, evidenced by the creation of multidisciplinary teams that ensure continuity of care and the establishment of community mental health centres offering a full range of services including day centres, crisis intervention, and home visits [55]. In recent years, it is possible to identify arguments for the advancement of mental health deinstitutionalisation, such as professionals’ beliefs and attitudes consistent with recovery principles towards people with psychiatric disabilities [56], national laws that facilitate economic autonomy by promoting labour market integration [57], equal standing opportunities for participation of users and civil society organisations in the development of a mental health agenda [58]. However, from the outset of mental health deinstitutionalisation, the exclusion of people with psychiatric disabilities from full citizenship has not been completely tackled. People with psychiatric disabilities are often physically present in the community but are still not fully included in it [59, 60]. Some of the factors that structurally maintain people with mental health challenges within a persistent social exclusion are associated with poverty (e.g., low-income social benefits or pensions), restricted access to valued social roles (e.g., employees, tenants, students, spouses, or parents), denial of legal capacity (e.g., property or income management) and long-term psychiatric confinement [17, 39, 61–65]. Mental health service users typically continue to live under professionals’ supervision and control regarding daily routines and medication protocols, with similar restrictions of an in-ward life, as in the old asylums [19, 66].

The United Nations Special Report on the Promotion and Protection of Human Rights on Mental Health [67] calls for a human rights approach to social change and transformation of how mental health is understood. The UN Committee on the Rights of Persons with Disabilities [1] on deinstitutionalisation emphasises the right to live independently and be included in the community. Rosenthal [68, p.5]] particularly argues, reflecting on death rates resulting from the COVID-19 pandemic, that psychiatric hospitals and other congregated mental health institutions “have typically been a dead end for millions… they have now become deadly incubators for the virus”, reinforcing the contemporary urgency of deinstitutionalisation in mental health.

This scoping review aims to contribute to the understanding of why, after over half a century, deinstitutionalisation has not yet been fully implemented to redirect resources and services towards community inclusion. For that purpose, we explore empirical research to identify evidence of what hinders and drives the deinstitutionalisation of adults with psychiatric disabilities. We adopt the system change framework by Foster-Fishman et al. [69] to categorise the identified hindrances and drivers into the fundamental explanatory elements. This system change framework, considered pertinent for an in-depth understanding of transformative changes in community mental health [70], recognises four fundamental dimensions in a system: (1) norms–beliefs, attitudes, and values that shape behaviour and generate a dominant normative, influencing practices and functions within a system; (2) resources–human, social, economic, and opportunity, enacting the purpose of the system, such as human capital, support services, and distribution of funds; (3) regulations–governmental rules or organisational policies that standardise and regulate behaviour and clarify of what is normative, expected, sanctioned and rewarded; and (4) operations–power and decision-making, regarding who controls the access to resources, who has the power, which seats or decision-making structures are required to change [69]. This scoping review seeks to shed light on the incomplete human right to deinstitutionalisation and life in the community while systematising scientific knowledge to inform mental health policymaking.

A scoping review is considered an adequate method to cover a wide body of literature, provide an overview of available evidence, and identify research gaps [71, 72]. Moreover, focusing on empirical studies provides evidence for the validity of research [73].

A preliminary search of PROSPERO, the Cochrane Database of Systematic Reviews, Open Science Framework, and JBI Evidence Synthesis was conducted. From our search, we have identified two reviews: a systematic review that focuses on evidence from the transition experience of patients into the community [74] and a published scoping review with some similarities concerning barriers and facilitators of psychiatric deinstitutionalisation [75], comprising studies from 1977 to 2019 and including all types of sources (grey literature, textual papers, reports, reviews, theoretical and political discussions). Building on these previous compilations of deinstitutionalisation literature, the current study seeks to update, strengthen, and advance the scientific knowledge of empirical evidence available in different electronic databases, from 1991 to 2024. In addition to the authors’ knowledge, no other review has been developed or proposed on what empirically hinders and drives mental health deinstitutionalisation for adults with psychiatric disabilities.

## Method

### Aim

This scoping review aims to identify which factors hinder and drive the deinstitutionalisation process of adults diagnosed with mental illness present in empirical peer-reviewed published studies, in a global perspective, from 1991 until 2024.

### Study design

The present study followed the Joanna Briggs Institute methodology for scoping reviews [72]. Our scoping review protocol was previously registered in Open Science Framework (Registration DOI: 10.17605/OSF.IO/QJAZE). The general objective for this scoping review was to explore and map what empirically hinders and drives the deinstitutionalisation of adults with mental illness. The PRISMA-ScR (Preferred Reporting Items for Systematic Reviews and Meta-Analyses Extension for Scoping Reviews) was used to report the results [76].

### Search strategy

A search strategy was developed with the supervision of two specialised librarians. An initial search of PubMed was undertaken to identify articles on the topic. The text words contained in the titles and abstracts of articles deemed relevant, and the index terms used to describe the articles, were used to develop a full search strategy based on the Peters et al. [72] PCC (Population, Concept and Context) model for scoping reviews. In our case, terms regarding adults with mental illness (population), deinstitutionalisation process (concept), and psychiatric hospitals and community mental health (context) were inserted in the search (see Table 1). After a preliminary assessment of adequate electronic databases, the following databases were selected for the full search strategy: PubMed, APA PsycINFO (via EBSCO), Web of Science, and Scopus (see Table 2). The antonym of mental health deinstitutionalisation, namely “institutionalisation” was included to capture the absence or loss of the concept. Only peer-reviewed articles published in English were included. Regarding the year of publication, only studies from 1991, since the United Nations’ adoption of the principles for protecting persons with mental illness and improving mental health care, until 2024, were included. Authors of relevant papers were contacted to request full-text access when considered necessary. The reference list of relevant articles was also hand-searched for additional papers.

**Table 1:** Search terms

Populations	Concept	Context
“mental health”	Deinstitutionalisation	“psychiatric hospitals”
“mental disorders”	Institutionalisation	“community mental health”
“mentally ill”		
“inpatients”		
“mental illness”		
“psychiatric disabilities”

**Table 2:** Search string

Database	Search string
PubMed	TI/AB (“mental health”[MeSH Terms]) OR TI/AB (“mental disorders”[MeSH Terms]) OR TI/AB (“mentally ill”[MeSH Terms]) OR TI/AB (“inpatients”[MeSH Terms]) AND TI/AB (deinstitutionalisation[MeSH Terms]) OR TI/AB (institutionalisation[MeSH Terms]) OR TI/AB (“community mental health services”[MeSH Terms])
APA PsycINFO	TI/AB (MH “mental disorders” OR MH “mental health” OR MH “mental illness”) AND TI/AB (MH deinstitutionalisation)
Scopus	TI/AB (“mental disorders” OR “psychiatric disabilities”) AND TI/AB (deinstitutionalisation OR institutionalisation OR “community mental health”)
Web of Science	TS=(“mental disorders”) OR TS=(“psychiatric disabilities”) AND TS=(“deinstitutionalisation”) OR TS=(“institutionalisation”) OR TS=(“community mental health”) OR TS=(“psychiatric hospitals”)

### Inclusion and exclusion criteria

#### Type of study

This scoping review considered peer-reviewed published articles that use a qualitative, quantitative, or mixed-method study design. Only empirical studies were included. Commentaries, descriptive reports, editorials, opinion papers, theoretical analyses, review studies, and grey literature were excluded.

#### Population

The present study concerns adults (age ≥ 18) diagnosed with mental illness who went through the deinstitutionalisation process. The population may also include mental health professionals, managers, family caregivers, key stakeholders, and other relevant data sources concerning the deinstitutionalisation of adults. Studies exclusively concerning the deinstitutionalisation of children or elderly people and studies that only concern the deinstitutionalisation of different population groups rather than people with mental health lived experiences were excluded.

#### Concept

This study addresses the concept of mental health deinstitutionalisation of adults with mental illness. Deinstitutionalisation is here defined according to the latest Guidelines on Deinstitutionalisation, Including in Emergencies by the United Nations Committee on the Rights of Persons with Disabilities, which describes deinstitutionalisation as a process of restoring independence, choice, and control to persons with disabilities, along with ending all forms of institutionalisation [1]. This UN 2022 document recognises institutionalisation as any form of violence, characterised by isolation and segregation, lack of choice and control, a rigid routine, and a paternalistic approach, where people with disabilities are deprived of living a free life in the community.

#### Context

The selection criteria of contributions addressing mental health deinstitutionalisation research resulted from a careful analysis of the nuances of the transition processes from different settings, including psychiatric hospitals, public or private inpatient services, or even other mental health clinical services in the community. The included literature was not limited to geographic location, nationality, or country gross national income.

### Screening and selection of evidence

Following the search, all identified records were collated and updated to Zotero software (www.zotero.com), and duplicates were removed. The created reference list of studies was screened for relevance. Titles and abstracts were screened independently by two research team members (LSF, JP) for assessment against the defined inclusion criteria. Potentially relevant papers were fully assessed independently against the inclusion criteria by the three members of the research team (LSF, JP, MJVM). Reasons for the exclusion of full-text papers that did not meet the inclusion criteria are recorded and reported in the scoping review. The disagreements between reviewers were resolved through discussion and with the input of a fourth highly specialised researcher (JHO). The results of the search were reported in full and presented according to the PRISMA extension for scoping reviews (PRISMA-ScR) flow diagram [76].

### Data extraction

The data selected from the included papers were extracted independently by three reviewers (LSF, JP, MJVM). A predetermined data extraction spreadsheet, developed by the reviewers in Microsoft Excel, was used following the research question. The data extraction tool comprises the author’s name and year of publication, the location where the study takes place, the aim of the study, the study setting, method, measures and protocols used in the study, the study population and sample size, and the empirical outcomes or findings concerning what hinders or drives mental health deinstitutionalisation. This tool was independently pilot-tested by the reviewers (LSF, JP, MJVM) through regular meetings to refine the overall extraction process. For the present scoping review, the concepts of hinder and driver were defined according to the online Oxford English Dictionary. Hinder is defined as “to keep back, delay, or stop an action; to put obstacles in the way of; to impede, deter, obstruct, prevent”; a driver is defined as “one of the main things that influence something or cause it to make progress”. Disagreements concerning data extraction were resolved through discussion among all authors.

### Analysis and presentation of results

Descriptive synthesis was used to present the results by summarising the characteristics of included studies (Table 3), identifying hindrances and drivers, and organising them into predominant categories. The extracted hindrances and drivers were compiled into two independent documents and synthesised independently by two members of the research team (LSF, BS). Patterns and codes were identified, and categories were defined according to the commonalities between the identified hindrances and drivers. The two research members met to identify the coherence between the two analyses, consulting a third research member (MJVM) for a final consensus. Finally, the identified categories were sorted according to the fundamental elements of the system change framework (norms, resources, regulations, and operations) [69].

**Table 3:** Characteristics of included studies

Ref.	Authors (year)	Country	Aim	Setting	Method	Participants/Sample	Measures and Protocols
[77]	Baltazar et al. (2013)	Brazil	How the habitus contributes to or restrains the social (re)integration after deinstitutionalisation	Inpatient discharge accommodations • Therapeutic residential services • People living alone	Qual	*n* = 4 people with serious mental illness	Ethnographic participant observation
[78]	Bennetts et al. (2011)	Australia	Explore consumer participation from a manager’s perspective	Mental health services • Public vs. private services • Inpatient vs. community services	Qual	*n* = 7 senior mental health managers	Semi-structured interviews
[79]	Bhugra et al. (2018)	Cross-national	To compare mental health policies to WHO’s standards for mental health policy and assess compliance with international recommendations	Policies and governance • Mental health policies • Mental health funding	Qual	*n* = 25 commonwealth countries’ mental health policies	WHO’s Mental Health Policy Checklist survey
[80]	Bosi et al. (2014)	Brazil	Explore the perspective of community workers on the relationship between social determinants and deinstitutionalisation	Community mental health • Psychosocial activities	Qual	*n* = 24 mental health community workers	Case study (focus group and interviews)
[81]	Braddock (1992)	USA	To test empirically the different roles of civil rights in the mental health and mental retardation fields and consumer advocacy impact on public spending	Policies and governance • Public spending on mental health	Quant	State government budget data from 1981, 1983, 1985, 1987	Five-factor hierarchical regression
[82]	Broulikova et al. (2020)	Czech Republic	To analyse mental health expenditures in the Czech Republic	Policies and governance • Mental health expenditures	Quant	Annual state mental health expenditures	OECD methodology on health accounts
[83]	Carbonell Marqués and Navarro-Pérez (2019)	Spain	To find out the extent of family care responsibility in mental health and to explore the structural barriers of the mental health care model from a professional psychosocial perspective	Mental health system • Family care • Psychosocial services	Qual	*n* = 37 mental health professionals	Focus group. In-depth interviews
[84]	Ceccherini-Nelli and Priebe (2007)	Cross-national	Explore the economic factors and the number of hospital psychiatric beds	Psychiatric services • Psychiatric hospital beds	Quant	CPI, real GDP, discount rate, rate of unemployment and psychiatric beds from USA, UK, Italy	Time series analytical technique
[85]	Chinman et al. (2001)	USA	Preliminary analyses and evaluation of a consumer-run peer-support-based programme (Welcome Basket)	Consumer-run services • Peer-support-based programmes	Quant	*n* = 158 (*n* = 79 Welcome Basket Participants and *n* = 79 Community Mental Health Centre Outpatients)	Two times comparative ANOVA
[86]	Chopra and Herrman (2011)	Australia	Assess the long-term outcomes for the original cohort of residents in the community care unit after 8 years of being discharged	Community mental health • Residential psychiatric rehabilitation unit	Mix	*n* = 18 mental health patients	Interviews and quantitative analyses of data from case records
[38]	Chow et al. (2019)	Cross-national	Explore the perspectives of mental health professionals on what has driven change in mental health reforms and deinstitutionalisation since 1990	Mental health system • Institutionalise care	Qual	*n* = 24 mental health professionals from England, Germany, Italy	In-depth interviews
[87]	Cubillos et al. (2020)	Cross-national	Present an overview of current policies concerning mental health reform and employment of people with severe mental illness	Community mental health • Supported employment programmes • Community rehabilitation services	Qual	*n* = 36 stakeholders from Colombia, Peru, and Costa Rica’s mental health systems (government officials, medical providers, patients, academics, and health providers)	Semi-structured interviews and public records analysis
[88]	Darcis et al. (2022)	Belgium	Analyse mental health reform and implementation of three policy plans	Policies and governance • Policy implementation instruments	Qual	*n* = 85 stakeholders (policymakers, coordinators, and network members); 77 meetings were observed	Case study; documents analysis; semi-structured interviews, non-participatory observation
[59]	Davidson et al. (1995)	USA	Understand, by the voice of long-stay inpatients, their experiences of returning to the community following discharge from a state hospital	Inpatient discharge accommodations • Long-stay inpatient services • Community mental health initiatives	Qual	*n* = 12 long-stay inpatients	In-depth interviews
[89]	Drake (2014)	Australia	Explore how boarding houses operate as an accommodation option within the policy of deinstitutionalisation	Inpatient discharge accommodations • Boarding houses	Mix	*n* = 40 participants (*n* = 10 residents; *n* = 15 staff; *n* = 12 government agencies; *n* = 3 proprietors)	Semi and in-depth interviews and document analysis
[90]	Duhig et al. (2017)	Australia	Examine readmission from the service users’ perspectives	Psychiatric services • Psychiatric Hospital	Qual	*n* = 13 psychiatric patients	Cross-sectional exploratory study interviews Grounded theory
[91]	Forchuk et al. (2008)	Canada	Test an intervention (assistance in finding affordable housing and community funds that cover the first and last month’s rent) following psychiatric admission	Inpatient discharge accommodations • Homelessness shelters • Supported apartment and independent housing	Mix	*n* = 14 patients with serious mental illness (*n* = 7 control group, *n* = 7 intervention group)	Randomised control trial and interviews
[92]	Gerson and Rose (2012)	USA	Explore perceptions of patients and families of patients’ needs, functioning, coping, and social support in the first 4 weeks after inpatient treatment	Inpatient discharge accommodations • Inpatient psychiatric unit • Family home in the community	Mix	*n* = 10 patients with serious mental illnesses	Two-time statistical analysis and interviews
[93]	Gulcur et al. (2003)	USA	Evaluate a housing first programme for formerly homeless people who resided in psychiatric hospitals immediately before study entry and compare it to programmes in the continuum of care	Inpatient discharge accommodations • Congregate living spaces • Independent housing (housing first programme)	Quant	*n* = 225 (*n* = 157 from the streets and *n* = 68 from two state psychiatric hospitals with major mental illness)	Randomised control trial
[94]	Hasson-Ohayon et al. (2016)	Israel	Understand the transition phase from psychiatric hospitalisation back to the community to learn from the personal life stories of people diagnosed with schizophrenia	Inpatient discharge accommodations • Psychiatric rehabilitation agency • Outpatient unit	Qual	*n* = 15 people diagnosed with schizophrenia	Semi-structured interviews and Narrative analysis using IPA.
[95]	Hobbs et al. (2002)	Australia	Six-year evaluation to assess the progress of participants from community and hospital-based residents	Inpatient discharge accommodations • Community living • Community care	Mix	*n* = 47 patients with long-term mental illness	Semi-structured interviews and two-time analysis of clinical measures
[96]	Hope et al. (2023)	Norway	Explores the places and new landscape created by the shift in mental healthcare from total institutions to a more community-based approach	Inpatient discharge accommodations • Places of living in the community	Qual	*n* = 13 participants with mental health and/or substance abuse issues	Walking interviews and stepwise-deductive inductive method
[97]	Howard et al. (2024)	Australia	Explores recent transition from institutional to community living from large residential centres	Inpatient discharge accommodations • Small group homes	Qual	*n* = 35 persons (*n* = 24 family members; *n* = 11 staff)	Interviews and group meetings
[44]	Hudson (2016)	Cross-national	Understand the drivers of the deinstitutionalisation of psychiatric care on an international basis	Psychiatric services • Psychiatric hospital beds	Quant	No. of psychiatric beds from 161 nations	Secondary regression analysis of existing data derived from WHO’s Atlas (2001, 2006, 2011, 2014, and other sources)
[52]	Jenkins et al. (2007)	Russia	Analyse mental health reform in one Russian oblast (region) using systematic approaches to policy design and implementation	Mental health system • User and non-governmental organisation care • Urban, semi-urban, and rural area • Mental health policies and funds	Mix	*n* = 200 (*n* = 46 generalist physicians; *n* = 93 mental health workers; *n* = 53 municipal social workers. *n* = 8 NGO workers)	Action research with document analysis, situation assessment, two-time analysis; questionnaire, no. of beds, admissions, and utilisation of services.
[98]	Jensen et al. (2010)	Canada	Assess a new model of discharge care, community-based discharge planning	Community mental health • Psychiatric hospital • Community-based discharge service	Mix	*n* = 36 people with serious mental illness	Data analysis and questionnaires
[99]	Jung et al. (2024)	South Korea	Explore housing policies and spatial characteristics of residential facilities and independent housing for individuals with psychiatric disabilities	Inpatient discharge accommodations • Residential facilities • Independent housing	Qual	*n* = 7 residential facilities (group homes; halfway houses; living facilities; independent housing)	Case analysis
[100]	Kamis-Gould et al. (1999)	USA	Examine the impact of closing a state psychiatric hospital on service utilisation patterns and related costs for clients with and without serious mental illness	Mental health system • Psychiatric hospital • Community services • Policies and funds	Quant	*n* = 2240 clients discharged from inpatient care	Cohort study cross-sectional and longitudinal perspective
[101]	Lee et al. (2012)	USA	Examine hospital effects and mental health funding on the length of psychiatric hospitalisation	Psychiatric services • Mental health funding	Quant	Records from 45,497 adults with serious mental illness discharged from 106 psychiatric hospitals	Descriptive and hierarchical linear modelling
[102]	Lora et al. (2020)	Cross-national	Describe the availability and delivery of mental health services in the WHO’s Member States, focusing on countries’ income levels	Mental health system • Mental health funding • Mental health services	Quant	*n* = 171 experts from WHO Member States	WHO Atlas questionnaire
[103]	Macdonald et al. (2018)	United Kingdom	Examine how the experience of deinstitutionalisation has impacted the lived experience of service users/survivors from their perspective	Inpatient discharge accommodations • Community care • Residential Care	Qual	*n* = 16 participants (*n* = 9 service users/survivors; *n* = 3 family members; *n* = 4 mental health practitioners)	Biographical narrative approach
[104]	Mazor and Doron (2011)	Israel	Understanding the meaning of returning to the community of persons suffering from schizophrenia while receiving a basket of social services from the Community Rehabilitation Act	Inpatient discharge accommodations • Community rehabilitation • Independent living • Mental health law	Qual	*n* = 15 adults with schizophrenia	Interviews
[105]	Miller and Rees (2014)	England	Explore change within the commissioning of third-sector mental health services and understand if commissioning has promoted change	Policies and governance • Third-sector organisations • Clinical commissioning groups	Qual	*n* = 29 participants (*n* = 23 third-sector organisation workers, *n* = 6 commissioners	Case study: online survey; semi-structured interviews
[106]	Moxham (2016)	Australia	Understand the housing environments inhabited by people with mental illness in the community following a period of institutionalisation and if these environments are ameliorated or contribute to feelings of stigmatisation and lack of control	Inpatient discharge accommodations • Community accommodations • Family home	Qual	*n* = 15 participants living with a serious mental illness	Interviews
[107]	Mulvale et al. (2007)	Canada	Examine how the legacy policies influence the development of a consumer-centred mental health system and rebalance spending from institutional to community care	Policies and governance • Mental health policies • Mental health spending	Qual	*n* = 17 key informants	Case study approach document analysis and interviews
[108]	Mutschler et al. (2022)	Canada	Examine the relationship between community integration, social support, and personal recovery following discharge from an inpatient hospital	Consumer-run services • Peer-support-based programmes	Quant	*n* = 72 Individuals with schizophrenia diagnoses	Secondary analysis of a phase 2 clinical trial; hierarchical regression and mediation analyses
[109]	Newton et al. (2000)	Australia	Evaluate the effects of deinstitutionalisation on the lives of individuals pre- and post-discharged	Inpatient discharge accommodations • Psychiatric hospital • Community residential facilities (large-style group homes)	Qual	*n* = 47 patients transferred to the community	Ethnographic approach participatory observation, open-ended and semi-structured interviews, and records.
[110]	Oh et al. (2022)	South Korea	Explore continuity of care following deinstitutionalisation	Community mental health • Inpatient services • Community-based services	Qual	*n* = 21 people diagnosed with schizophrenia	In-depth interviews
[111]	O’Shea and Williams (2023)	Australia	Evaluate the Pathways to Community Living Initiative from the perspective of service users and family members	Inpatient discharge accommodations • Psychiatric hospital • Community living (care facilities, group homes, public housing)	Qual	*n* = 39 respondents (*n* = 27 service users; *n* = 12 family members)	Semi-structured interviews
[112]	Phehlukwayo and Tsoka-Gwegweni (2018)	South Africa	Investigate how contextual factors influenced care coordination for chronic mental illness care within the eThekwini District	Community mental health • Community health centres • Community support services	Qual	*n* = 48 key informants (*n* = 3 Psychologists, *n* = 30 community caregivers, *n* = 8 nurses, *n* = 4 occupational therapy, *n* = 2 social workers)	Semi-structured interviews
[113]	Pilisuk (2001)	USA	How independent living programmes may affect the quality of supportive networks among formerly hospitalised psychiatrically disabled population	Inpatient discharge accommodations • Social rehabilitation programme • Independent living	Qual	*n* = 47 people formerly psychiatric hospitalised (*n* = 25 people living independently)	Interviews
[114]	Pinfold (2000)	England	To explore the positions, roles, and therapeutic benefits established by socio-spatial networking in the community through the experiences of people with enduring mental health problems	Community mental health • Community living • Rehabilitation services	Qual	*n* = 39 participants (*n* = 25 services users with serious mental illness, *n* = 14 mental health professionals)	In-depth interviews
[115]	Pinkney et al. (1991)	Canada	Evaluate the effects of the transition from hospital to community on people discharged from rehabilitation programmes, focusing on their quality of life in community settings	Inpatient discharge accommodations • Hospital-based rehabilitation programmes • Community living	Mix	*n* = 55 people discharged from inpatient psychiatric rehabilitation programmes	Interviews and progress measures
[116]	Quah (2017)	Singapore	Identify and discuss the main barriers to partnership between family caregivers and the medical team in the context of de-institutionalisation	Mental health services • Family caregivers • Medical teams	Mix	*n* = 47 Family Caregivers of people diagnosed with schizophrenia	In-depth interviews, structured questionnaires, and attitudinal scales
[117]	Salisbury et al. (2016)	Cross-national	Develop a quantitative measure of country-level progress towards deinstitutionalisation and comparison of deinstitutionalisation progress	Mental health system • Mental health policy	Quant	Data from 30 European Countries reported by experts	Creation and validation of a mental health deinstitutionalisation measurement tool.
[118]	Sampaio and Bispo Junior (2021)	Brazil	Understand mental health care offered by RAPS (Psychosocial Attention Network)	Community mental health • Community psychosocial services	Qual	*n* = 33 stakeholders (*n* = 5 MH service users, *n* = 21 health professionals, *n* = 7 policymakers)	Semi-structured and in-depth interviews
[119]	Sather et al. (2019)	Norway	Explore former patients’ views of pathways in transition between district psychiatric hospital centres and community mental health services	Mental health services • Psychiatric Hospitals • Community and rural mental health activity centres	Qual	*n* = 10 former inpatients mental health	Interviews
[120]	Sealy and Whitehead (2004)	Canada	Empirically trace the extent to which deinstitutionalisation has been achieved, whether it has been uniform, complete, or just transinstitutionalisation	Psychiatric services • Mental health policy • Psychiatric hospital beds • Psychiatric services expenditures	Quant	The number of psychiatric beds; days of care; average length; expenditures	Comparison of standardised population-based rate measures
[121]	Shek and Pietila (2016)	Russia	Analyse the Russian mental health policy reforms from the outpatient mental health practitioners’ viewpoint	Community mental health • Outpatient mental health clinics	Qual	*n* = 33 outpatient specialist professionals	Interviews
[47]	Shen and Snowden (2014)	Cross-national	Empirically examine whether the institutionalisation of deinstitutionalisation policy changed the supply of psychiatric beds in 193 countries from 2001 to 201	Mental health system • Mental health policies • Psychiatric hospital beds	Quant	Deinstitutionalisation policy adoption, no. of mental hospital beds, general hospital beds, and all psychiatric beds from 193 countries	Random effects linear models
[65]	Shen et al. (2017)	Cross-national	Investigate the norms, actors, and strategies that influence the uptake of deinstitutionalisation internationally	Mental health system • Hospital-based care • Community-based care	Qual	*n* = 78 experts representing 42 countries	Survey designed to compare deinstitutionalisation across countries
[122]	Smith et al. (2021)	Belgium	Understand stakeholders’ coalitions, why reforms are lagging, and what would help to coordinate policy implementation more effectively	Policies and governance • Mental health policy • Stakeholders’ coalitions	Quant	*n* = 469 Stakeholders (policymakers, service managers, clinicians, and user representatives)	Questionnaire and ANOVA
[123]	Stoeckel et al. (2022)	Serbia	Determine the quality of life in the domain of social belonging/community integration, in the differences between persons with ID/MHP living in institutional residences and community settings, and the service provider (governmental/state-run or non-governmental sector)	Inpatient discharge accommodations • Institutional residence (Halfway houses) vs. non-institutional community settings • Governmental/state-run services vs. non-governmental organisation services	Quant	*n* = 71 persons with intellectual disability and mental health problems that were deinstitutionalised	Comparative study of the application of the quality-of-life questionnaire
[124]	Topor et al. (2016)	Sweden	Examine the institutional landscape that has emerged in a Nordic welfare state after the closing of psychiatric hospitals	Mental health system • Social welfare agencies • Psychiatric care • Prisons	Quant	*n* = 1355 persons with a diagnosis of psychosis	Naturalistic prospective study of socio-demographic data from secondary sources
[125]	Wiktorowicz (2005)	Canada	Understand why community sector reforms have not kept pace with institutional downsizing	Policies and governance • Mental health policy	Qual	*n* = 19 key informants	Document analyses and semi-structured interviews
[126]	Williams et al. (2023)	Australia	Independent evaluation of the transitioning from hospital and practice change in mental health services	Inpatient discharge accommodations • Psychiatric hospital • Community living (care facilities, group homes, public housing)	Mix	*n* = 1004 people diagnosed with mental illness	Kessler Psychological Distress Scale; Health of the Nation Outcome Scales; Life Skills Profile; Resource Utilisation Groups; Semi-structured interviews
[127]	Winkler et al. (2018)	Czech Republic	Compared quality of life and societal costs in people with psychosis who had been receiving care in psychiatric hospitals versus those who had been discharged to community care	Policies and governance • Mental health expenditures	Quant	*n* = 115 patients (*n* = 35 of community services and *n* = 80 of inpatients)	Questionaries regarding quality of life and Cost-effectiveness

## Results

### Search results

**Fig. 1:**
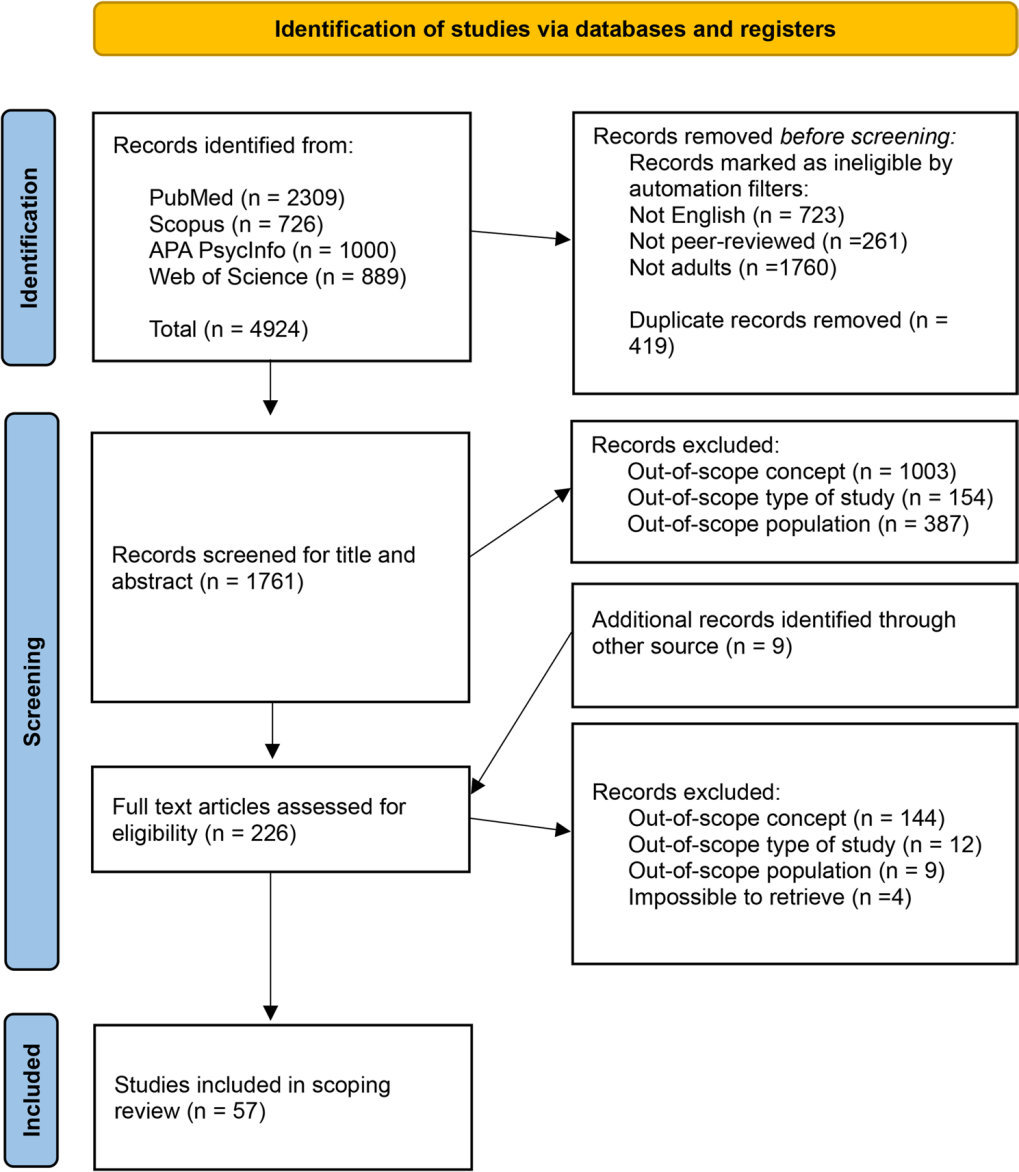
PRISMA flow chart of study selection

The full database search, conducted between January and March 2024, generated a total of 4924 references from the following four databases: PubMed (*n* = 2309), Scopus (*n* = 726), APA PsycInfo (*n* = 1000), Web of Science (*n* = 889) and from additional records identified by screening the reference list of relevant studies (*n* = 9). Before the screening, duplicate records (*n* = 419) and records that did not fit the inclusion criteria using database automatic filters, such as English, adult, and peer-reviewed articles (*n* = 2744), were removed. A total of 1761 references were considered for title and abstract screening against inclusion criteria. Records with the out-of-scope concept (*n* = 1003), out-of-scope type of study design (*n* = 154), or out-of-scope population (*n* = 387) were excluded, leaving a total of 226 eligible articles for further assessment. After the full-text screening, another 169 articles were excluded by being out of scope in terms of concept (*n* = 144), type of study design (*n* = 12), population (*n* = 9), and additionally for being impossible to retrieve (*n* = 4). A final selection of 57 studies was included in the scoping review (see Fig. 1).

### Characteristics of included studies

The 57 included studies were organised according to alphabetical order of first authors’ last names. Regarding study design, most studies (53%; *n* = 30) employed a qualitative approach, primarily using interviews. In contrast, 30% (*n* = 17) had a quantitative design, which included cross-sectional or longitudinal designs, and 17% (*n* = 10) utilised a mixed-methods study design. The higher percentage of the studies (26%) were conducted in the North Americas (*n* = 15), 25% were conducted in Europe (*n* = 14), 17% were conducted in Oceania (*n* = 10), 9% were conducted in Asia (*n* = 5), 5% were conducted in South America (*n* = 3), and 2% were conducted in Africa (*n* = 1). Moreover, we included nine cross-national studies corresponding to 16% of the overall pieces selected for analysis. Out of the nine cross-national studies, one concerned North and South American countries, another concerned 25 Commonwealth member countries, three studies focused on Western and European countries, and the remaining five cross-national studies ranged from 42 to 193 countries from all over the world and from varied country income profiles. Regarding the years of publication, the publication dates of the included studies ranged from 1991 to 2024. Most of the studies (60%) were published from 2014 to 2024 (*n* = 34), 24% were published between 2002 and 2013 (*n* = 14), and 16% were published between 1991 and 2001 (*n* = 9). Concerning the type of sample, out of the total 57 studies, 45% included adults with mental illness (*n* = 31), 21% of the studies included mental health professionals or managers (*n* = 14), 15% used secondary data sources and document analysis (*n* = 10), 13% included other stakeholders (e.g., policymakers, experts, or commissioner; *n* = 9), and 6% of the studies included family caregivers of adults diagnosed with mental illness (*n* = 4). Regarding study settings, most studies (35%) concerned inpatient discharge accommodations, the settings to which people transition from inpatient psychiatric services (*n* = 20); 16% of the studies were associated with policy and governance, including legislative, policy organisation, and funding settings (*n* = 9); 16% concerned community mental health settings such as rehabilitation centres, community programmes and services (*n* = 9); another 16% included macro level of analysis, regarding the mental health system as a whole, including the institutional, family and community care (*n* = 9); 9% (*n* = 5) of the studies were conducted in psychiatric services settings (e.g., psychiatric hospitals); 5% addressed a multi-level analysis of public, private, inpatient and community mental health service settings; and 3% of the included studies were held in consumer-run services, such as peer-support programmes (*n* = 2). Table 3 describes the features of the included studies.

### Review findings

#### [[Hindrances and drivers

Through the scoping of the 57 empirical studies, it was possible to map 129 hindrances out of 40 studies (see Appendix 1) and 89 drivers in 37 studies (see Appendix 2) regarding the deinstitutionalisation of adults with mental health challenges. These findings were then compiled into main categories and organised into four fundamental sections guided by the system change framework [69] (see Table 4.). In the following result section, we describe each category.

**Table 4:** Mental health deinstitutionalisation hindrances and drivers

**System Elements**	**Key Categories of Hindrances**	**Ref**	**Key Categories of Drivers**	**Ref**
Norms	The exclusiveness of the medical model	[47, 52, 78, 83, 88, 89, 97, 103, 105, 107, 115, 117, 118]	Model for community inclusion	[59, 80, 95, 96, 99, 108, 109, 118]
	Social discrimination	[110, 114, 121].	Inclusive society	[44]
Resources	Insufficient community services	[38, 52, 83, 86, 90, 92, 99, 103, 106, 110, 112, 117, 118].	Resourcing community alternatives	[44, 59, 91, 93, 94, 96–98, 100, 101, 109, 114, 118, 119, 123, 126]
	Transinstitutionalisation	[38, 47,77, 83, 86, 87, 89, 97, 99, 103, 106, 110, 115, 120, 123, 124].	Independent housing	[59, 77, 91, 93, 95, 99, 104, 113]
	Lack of support for community inclusion	[59, 83, 87, 90, 92, 97, 105, 106, 110–112]	Individualised context-oriented support	[59, 65, 86, 92–95, 97, 100, 108, 111, 114, 115, 118, 119, 126]
	Most funds allocated to institutionalisation	[52, 79, 82, 83, 87, 101–103, 120, 125]	Economic pressures	[38, 84, 93, 127]
	Economic incentives for institutionalisation	[38, 52, 101, 103, 107, 110, 120]		
Regulations	Institutional policies	[79, 87–89, 107]	Policy and legal reform	[38, 44, 47, 59, 65, 91, 99, 100, 104]
	Inefficient governance	[52, 79, 83, 105, 110, 118, 122, 125]		
Operations	Professional control	[47, 52, 65, 77, 78, 94, 106, 107, 116, 118, 119, 121, 123, 124]	Consumer participation in services	[59, 78, 85, 86, 97, 103, 119]
	Limited advocacy	[87, 125]	Consumer advocacy	[65, 81, 107]

### Hindrances of mental health deinstitutionalisation

#### Hindrances related to norms

##### The exclusiveness of the medical model

Across the studies, the medical model was identified as a hindrance to the advancements of mental health deinstitutionalisation [47, 52, 78, 83, 88, 89, 97, 103, 105, 107, 115, 117, 118]. The medical model is portrayed as a narrowed symptom-focused perspective of mental disorders [52, 78], centred on causes and symptom remission and medication adherence, rather than focusing on reintegration into society [83]. The prevalence of hospital-based mental health treatment and the lack of understanding of a person-centred, community-based, and well-being focus were pointed out as an obstacle to deinstitutionalisation [105, 107]. The dominance of the medical model reinforces the use of traditional institutional models and policy instruments that promote this prevailing logic in the mental health field [78, 88, 89, 97]. Notably, the legacy of psychiatric hospital policies and the hospital functioning in the current health care system tends to undermine social approaches, and it is identified as an inhibiting factor to mental health care system change [78, 89, 97, 103, 107]. Moreover, the significant reliance on hospital-based acute settings and inpatient psychiatric units as accommodation solutions contributes to centralised treatment practices. These practices are justified as user protection, maintaining people institutionalised and excluded from society, sometimes upon the person’s demise [78, 83, 115, 117, 118]. The medical model also influences staff custodial and paternalistic attitudes, and practices that rely on symptomatology, pressuring mental health professionals to oppose deinstitutionalisation [47, 52, 78].

##### Social discrimination

Although not as visible as other hindrances, social discrimination was identified as a relevant obstacle to deinstitutionalisation advancements [110, 114, 121]. The discrimination and prejudice of others and the societal stigma of not accepting mental illness experiences like other individual differences undermine the efforts to live and recover in the community [110, 114]. The social discrimination of the media, mainly the way people with this experience are depicted, also increases the negative representations in society and interferes with the deinstitutionalisation process [121].

#### Hindrances related to resources

##### Insufficient community services

Several studies reported that mental health deinstitutionalisation is hindered by shortcomings of community care following discharge [38, 52, 83, 86, 90, 92, 99, 103, 106, 110, 112, 117, 118]. Some studies identified the lack of community mental health professionals and lack of support, such as outreach teams, when transitioning to the community [83, 92, 103, 117]. Other studies acknowledged that the lack of housing choices, the lack of vacancies, and the unavailability of suitable accommodation options and social circumstances (e.g., societal malaise or access to resources incompatible with community tenure) as barriers to deinstitutionalisation [90, 99, 106, 115, 118]. Studies also found that a lack of quality community resources, such as civil society organisations and community resources that promote and support independent living, hinders the transition to the community [52, 83, 99, 106]. These shortcomings of community services lead to more investment in hospital beds and the creation of new forms of institutional care that increase the difficulty in maintaining community tenure, encourage family members to institutionalise, and drive the integration or re-institutionalisation into facilities away from family and friends [38, 86, 90, 99, 110].

##### Transinstitutionalisation

Most studies reported transinstitutionalisation as a hindrance to deinstitutionalisation; hence, the transition to community living often does not result in community inclusion. Instead, it frequently leads to transinstitutionalisation into smaller-scale congregated settings [38, 47, 77, 83, 86, 87, 89, 97, 99, 103, 106, 110, 115, 120, 123, 124]. Settings include group homes, therapeutic residences, rehabilitation centres, protected housing, boarding houses, hospital-based psychiatric rehabilitation programmes, and halfway houses [38, 77, 83, 86, 89, 115, 123]. These settings were found not to be appropriate housing solutions; rather, they were recreations of smaller-scale institutional structures, roles, and processes where people were still away from the community and their social support [89, 97, 99, 123]. Some of the reasons identified for the transinstitutionalisation phenomenon were associated with (a) investment in outdated segregated programmes, (b) inadequate planning for independent living before hospital discharge, (c) privatisation of healthcare, (d) the transference of an institutionalist culture to community services, and (e) a co-existing trend to re-institutionalisation [47, 87, 103, 110, 123]. Transition to these congregated settings was found to be restrictive of individual freedom, autonomy, and social integration, contributing to exclusion, marginalisation, and violation of human rights, therefore hindering deinstitutionalisation [77, 83, 86, 89, 106, 124]. This phenomenon is well illustrated in the phrase presented in Drake [89, p. 252]]: “Despite more than three decades of research, and a clear legislative and policy framework that people have the right to live in non-congregate accommodation, institutions still have a significant role in accommodating and “warehousing” people with mental illness and disability”.

##### Lack of support for community inclusion

The insufficiency of community services hinders the deinstitutionalisation process, as well as the lack of support that promotes community inclusion [59, 83, 87, 90, 92, 97, 105, 106, 110–112]. Following discharge, people are placed in community contexts; however, they lack the support to be part of specific community settings [59]. The absence of support services centred on people’s needs and the lack of promoters of a significant life and social connectedness with others undermine meaningful social inclusion [83, 90, 92, 105, 111]. Transitioning from inpatient wards to communities requires the deliberate enhancement of opportunities for community inclusion, which can be attained through structured programmes, such as supported housing and supported employment; nevertheless, the shortage of these services, poor employment support (e.g., unarticulated job training opportunities with employers), or support for social inclusion being a low priority of staff from residential centres are significant obstacles to deinstitutionalisation [87, 90, 97, 110–112]. Finally, deinstitutionalisation is also hindered by the lack of continuity of care [110], by housing provision and living support being separated services [97, 106], and the family’s lack of knowledge and support about recovery [110].

##### Most funds are allocated to institutionalisation

A low proportion of the health financial budget is spent on mental health care. This lack of funding for mental health hinders the development of the necessary resources for the advancement of deinstitutionalisation [79, 82, 83]. Moreover, it is also reported as an obstacle that most budget funds are absorbed by psychiatric hospitals, segregated programmes, and inpatient care, leaving few financial resources for community care [52, 82, 87, 101–103, 120, 125]. This inefficient distribution of funds is attributed to a lack of political will to allocate financial resources to community care due to historical expenditure procedures and the availability of infrastructures rather than prioritising the needs of people with psychiatric disability [52, 83, 102, 125].

##### Economic incentives for institutionalisation

Regarding financial resources, some studies identified the existence of economic incentives for the conservation of institutionalisation as a hindering factor [38, 52, 101, 103, 107, 110, 120]. Financial incentives for maintaining high bed occupancy in long-stay institutions and prioritizing inpatient treatments hinder efforts to transition people to the community [52, 110, 120]. Additionally, the growing privatisation of mental health care is driven by the increased economic interest in institutional care [38, 103]. Governmental or private health insurance systems also mainly cover and reimburse hospital services, functioning as a hindrance to accessing community services [101, 107].

#### Hindrances related to regulations

##### Institutional policies

In the included studies, it was possible to identify that countries’ lack of clear policies supporting community integration, recovery, and deinstitutionalisation hindered their advances [79, 87, 107]. Moreover, deinstitutionalisation is also deferred by policy agendas endorsing the use of institutional models of care, legitimising the “warehousing” of people with psychiatric disabilities in large-scale settings instead of focusing on evidence-based social programmes, like supported employment and housing [87–89]. Concerning policies, studies also reported the lower priority of mental health policies in comparison with physical health policies; the continued privileged position attributed to psychiatrists in policymaking over other mental health professionals hinders a broader public interest in mental health reform, delaying the deinstitutionalisation processes [107].

##### Inefficient governance

Some studies reported several obstacles to deinstitutionalisation regarding inefficiency in governance [52, 79, 83, 105, 110, 118, 122, 125]. Intersectoral collaboration among different political sectors (e.g., health, employment, housing, social welfare, education, and justice) is required to facilitate the transition to the community, concluding that the absence of intersectoral collaboration hinders deinstitutionalisation [52, 79]. Additionally, a lack of integrated mental health care, unclear roles, and a lack of trust between mental health services, primary care, and community care constitute barriers to deinstitutionalisation [83, 118]. Furthermore, divergencies, lack of collaboration, absence of a common healthcare framework among mental health providers, and diffuse regional authority mandates promoting deinstitutionalisation are holding back consistent advances [83, 105, 110, 122, 125].

#### Hindrances related to operations

##### Professional control

Across the empirical studies, it was reported that professional control hinders the advances of mental health deinstitutionalisation [47, 52, 65, 77, 78, 94, 106, 107, 116, 118, 119, 121, 123, 124]. Mental health service providers hold a position of power and privilege, which are misused to dominate and control people with mental illness, sometimes under the assumption that users need protection [77, 106, 107, 118, 121, 124]. Studies indicate that professional control is characterised by custodial and paternalistic attitudes, disincentive and bring about pessimism about recovery, and fosters hierarchical positionings between staff and user in decision-making, tending to exclude others in this clinical decision, such as the family members [52, 77, 94, 107, 116, 119, 121, 123]. Professional control excludes the focus on the person and the user power to participate in discharge plans meaningfully, enhances stigma, harms the identity of people with psychiatric disabilities, and restricts autonomy, choice, freedom, and community inclusion, constituting severe resistance and opposition to deinstitutionalisation [47, 77, 78, 94, 106, 119, 121].

##### Limited advocacy

The lack of advocacy groups representing people with mental illness in society and limiting people’s influence in political decisions were found in the scoped articles as hindrances of mental health deinstitutionalisation [87, 125].

### Drivers of mental health deinstitutionalisation

#### Drivers related to norms

##### Model for community inclusion

Many studies reported that shifting from an institutional to a community-based and citizenship model drives deinstitutionalisation [59, 80, 95, 96, 99, 108, 109, 118]. For a successful discharge, the transition to the community should be to regular settings and proximal community context in mainstream society [59, 108, 109]. A new model of care that values autonomy and social participation is open to the voices and lives of people with this experience, advances social needs instead of symptoms remission, creating the opportunity for people to live, work, study, and develop supportive relationships in the community [59, 80, 95, 96, 99, 108, 118]. The empirical results of the included studies showed that a community inclusion model for deinstitutionalisation has the potential to promote equality, income, social cohesion, and participation in policy, facilitate regaining freedom, choice capacity, and control over one’s own life while predicting social supports and recovery [59, 80, 108, 109].

##### Inclusive society

It was possible to identify, specifically in one study, that improvement of macro indicators of social justice characterises a more inclusive society and leads to the advancement of deinstitutionalisation. The study revealed that greater ethnic diversity, a higher Human Development Index, a higher Index of Democratisation, and a strong secular–rational culture relate to driving mental health deinstitutionalisation [44]. This is because, in more heterogeneous nations, there is less resistance to the inclusion of people after institutionalisation; more democratic societies and secular-rational cultures, rather than more traditional cultures, demonstrate more tolerance, generating more supportive and inclusive community services; furthermore, in democratic countries, separable goods and services prove important for a political base in which the community serves this purpose better than institutionalisation [44]. However, the same study also found that a higher imprisonment rate is associated with reduced hospital care [44].

#### Drivers related to resources

##### Resourcing community alternatives

A widely reported element in the scoped literature is the availability and development of community-based alternatives as a foundation for reducing reliance on institutional care [59, 93, 94, 96–98, 114, 118, 119, 123, 126]. Effective transitions from inpatient settings require robust, non-institutional services and community-based discharge planning to ensure continuity of care [44, 98, 123]. These alternatives should be rooted in local communities, tailored to individual needs and preferences, and focus on people’s strengths and capacities to engage fully in daily life. Such services support autonomy, participation in social activities, and the ability to lead diverse and fulfilling lives within the community [59, 96, 97, 114, 118, 119, 126]. A key component of successful community reintegration is collaboration with local resources, including non-governmental organisation service providers and individualised housing programs, such as Housing First. These partnerships enable individuals discharged from inpatient facilities to access stable housing, pursue employment opportunities, and engage meaningfully with the community, thereby reducing psychiatric institutionalisation and supporting lasting transitions [93, 123, 126]. In parallel, the allocation of financial resources directly to the community system plays a crucial role in enabling these changes [91, 100, 101, 109, 118]. Redirecting public funds from the closure of state psychiatric hospitals into community infrastructure [100, 101], alongside expanded financial support for independent living and sustained investment in high-quality community psychosocial services, has been shown to strengthen community care capacity and sustain the outcomes of mental health reform [91, 109, 118].

##### Independent housing

Concerning the transition process, studies singled out that deinstitutionalisation is driven by direct access to independent housing upon discharge [59, 77, 91, 93, 95, 99, 104, 113]. Independent housing consists of living by oneself, with no need to be house-ready, in a home in the community, in mainstream society, along with house and community inclusion support [59, 77, 91, 93, 99, 113]. Positive outcomes in housing transition at discharge have been emphasised by housing first programmes, experimental independent living programmes, immediate income, and support to access a household in the community. The studies found that independent housing promotes autonomy by enabling independence [77], choice [93], improvement in domestic skills [95], and less dependence on professionals [113]. Also, the smaller the scale of the house, the less is the necessity of supervision [95]. The studies also illustrate that social inclusion is promoted by independent housing with support, by facilitating community tenure [93], developing social networks [113], improving community skills [95], and creating a better opportunity to obtain employment [113]. Hence, independent housing enhances the capacity to live in the community, driving deinstitutionalisation [77, 91, 93, 95, 113].

##### Individualised context-oriented support

Many studies identified individualised supports provided to users in the community while addressing contextual barriers as drivers of mental health deinstitutionalisation [65, 86, 92–95, 97, 100, 108, 111, 114, 115, 118, 119, 126]. Detailed and personalised discharge planning, the availability of long-term and continuum community support, collaborative practice in the definition of care plans, working in partnership, and building bridges between mental health services, organisations, and individuals in the community were reported as significant professional support for an effective transition [65, 86, 95, 100, 111, 114, 115, 119]. Moreover, support should be individualised, tailored, and match people’s needs and interests related to community resources. This support is also a multilevel intervention, emphasising the role of community and family, besides meeting individual needs. It focuses on the social determinants of health, reducing contextual barriers, and on the creation of supportive communities rather than only adapting the individual [59, 86, 93, 94, 108, 114, 115]. This support contributes to an increased sense of belonging and positive identity, normalising social and recreational activities, supported living, supported employment, and supported education programmes in the mainstream community [59, 93, 94, 126]. Social support creates stability and community residence and predicts recovery after discharge. In this regard, advocating for deinstitutionalisation should focus on helping people build connections and develop personal support networks in the community fabric [59, 86, 108, 115]. Lastly, family members’ support is also considered fundamental and significant for transitioning to community living [92, 97, 111, 118].

##### Economic pressures

Economic factors play a significant role in shaping the landscape of mental health care and advancing the transition from institutional to community-based services. Periods of high inflation have been associated with a marked decrease in psychiatric bed availability, highlighting the sensitivity of inpatient care infrastructure to broader economic conditions [84]. This reduction is often influenced by political decision-makers who view deinstitutionalisation as a strategic response to the substantial costs of maintaining inpatient facilities, positioning community-based care as a more financially sustainable alternative [38]. Innovative community programs, such as the Housing First model, further exemplify the dual benefits of enhancing formerly institutionalised people outcomes and reducing public expenditure. By increasing housing tenure and decreasing reliance on psychiatric institutions, these programs present cost-effective solutions that alleviate economic burdens while supporting recovery and social integration [93]. More broadly, the transition to community care is widely recognised as a cost-effective approach, balancing improved quality of life for individuals with sustainable resource allocation within mental health systems [127]. Collectively, these economic considerations underscore how fiscal pressures and cost-effectiveness imperatives drive reforms that reshape mental health service delivery toward community-based alternatives.

#### Drivers related to regulations

##### Policy and legal reform

Favourable mental health policies and legal frameworks have been consistently identified as key drivers of deinstitutionalisation. The presence of mental health laws and regulations governing psychiatric commitment contributes to a significant decrease in the number of psychiatric beds [44, 47]. Furthermore, policies aimed at supporting community reintegration and independent living, particularly those addressing barriers to basic needs such as income and housing, play a crucial role in preventing homelessness and fostering community inclusion after discharge [91, 104]. In addition, broader mental health system reforms have been influenced by various policy modifications and legal changes that promote a shift from institutional to community-based care, from large-scale to small-scale facilities, and from treatment-centric approaches to models emphasising independent living support [99]. These reforms are often encouraged by the recognition of human rights violations, the deteriorating conditions in old asylums, and the overall poor state of psychiatric hospitals, all of which underscore the urgent need for systemic change [38, 47, 65]. Exogenous shocks such as war and natural disasters have also been found to inadvertently accelerate the reduction of psychiatric beds [47]. Crucially, the success of these reforms often depends on coordinated efforts across all levels of government and the application of research-based decision-making processes [47, 65]. Ultimately, the effective closure of psychiatric institutions signifies a tangible advancement in deinstitutionalisation practices [100].

#### Drivers related to operations

##### Consumer participation in services

As opposed to professional control over users and services, different forms of active participation of consumers were reported as enablers of deinstitutionalisation [59, 78, 85, 86, 97, 103, 119]. Consumers can participate as employed consultants by training and educating other mental health service providers, which, in turn, facilitates positive changes and fosters consumer power within the mental health system [78]. In addition, including the perspective of consumers and engaging in alternative approaches from the perspective of psychiatric survivors can lead to a mental health service focus on community inclusion, expose covered inequalities, and unveil where the old institutional model persists [86, 103]. Collaborative mental health professionals’ practices when moving to the community (e.g., sharing all information with consumers, ensuring that people have the freedom to choose, and increasing the control over their own lives) can also advance community living and prevent re-institutionalisation [59, 97, 119]. Moreover, participating in consumer-run mental health services at discharge or in peer-support programmes, solely staffed and managed by consumers, can help expand social networks and support the transition to the community [85, 119].

##### Consumer advocacy

Some studies found that consumer advocacy can further advance deinstitutionalisation [65, 81, 107]. Public figures speaking out on their personal experience of mental illness can lessen stigma and promote changes in society’s prejudiced ideas [107]. Non-governmental organisations advocating for mental health can be a crucial force, and consumers sharing their experiences in a way that resonates with community understanding can progress deinstitutionalisation [65, 81]. Finally, civil rights initiatives that are not weakened by internal competition but with strong common objectives can increase public funding and expand community resources [81].

## Discussion

This scoping review was able to identify the most predominant hindrances and drivers of the deinstitutionalisation process of adults diagnosed with mental illness. For this purpose, 57 empirical studies were reviewed, containing recent research findings from samples that encompassed experience from people diagnosed with mental illness, mental health professionals, family members, other stakeholders, documental analysis, and secondary data, as well as representing the six populated continents of the world. Hindrances included the exclusiveness of the medical model, social discrimination, insufficient community services, transinstitutionalisation, lack of support for community inclusion, most funds allocated to institutionalisation, economic incentives for institutionalisation, institutional policies, inefficient governance, professional control, and limited advocacy. Drivers included a model for community inclusion, an inclusive society, resourcing community alternatives, independent housing, individualised context-oriented support, economic pressures, policy and legal reform, consumer participation in services, and consumer advocacy.

Concerning the empirical research on mental health deinstitutionalisation of adults, this study revealed an affluence of studies conducted in Western and high-income countries. This phenomenon is possibly related to Western countries being the “early adopters” of deinstitutionalisation policies [47], thus producing a greater amount of empirical research on this topic. Low and middle-income countries also continue to be more anchored to inpatient care [128, 129]. Nevertheless, some global representation is assured by including 16% (*n* = 9) cross-national studies. Only four studies involved family members’ perspectives, which can be considered a gap in mental health deinstitutionalisation empirical literature because most people transitioning from psychiatric hospitals rely on family members’ support [74]. Concerning the setting of included studies, only two studies are held in consumer-run services, such as peer-support programmes. Considering that consumer participation and peer support are essential elements for mental health recovery and transformative change [54, 130], their low representativeness is also considered a gap in mental health deinstitutionalisation empirical literature. The remaining studies showed a rich representation of relevant samples, topics, and settings for the mental health deinstitutionalisation process. Regarding the categories identified, all of them present a variety of different types of samples, the continent where the study was conducted, and the years of publication of the scoped studies that constitute them. Only the category identified as Independent Housing was synthesised from scoped studies that contained only one type of sample, people diagnosed with mental illness. This could mean that the independent housing solution continues to be advocated mainly by the voice of consumers of mental health services [131]. Considering the heterogeneity of results, the following discussion focuses on the prevalent hindrances and drivers identified in mental health deinstitutionalisation. For a better understanding of continued hindrances and the potential root causes that have delayed deinstitutionalisation progress and to highlight the possible levers of change that drive deinstitutionalisation forward, we framed our findings into the four fundamental elements of the system change framework developed by Foster-Fishman et al. [69].

Deinstitutionalisation is not a mere reduction of psychiatric beds and the closure of segregationist structures; it is also an ideology, a social movement that demands the community integration of people with lived experience of mental illness in society [132]. To this end, it is essential to recognise that the deinstitutionalisation processes have not necessarily led to community inclusion [133, 134]. Some argue that it is due to the vague meaning of community in policies, research, and practices, resulting in a deviation from what was first intended for community mental health. Instead, social justice and human rights should be specified and at the centre of political discussions [135]. In this sense, our results intend to contribute to this premise by identifying what norms, resources, regulations, and operations hinder and drive the deinstitutionalisation processes of adults diagnosed with mental illness into being a part of the community.

Systems norms relate to the attitudes, values, and beliefs of stakeholders. They are the “deep structures” that demonstrate how the system operates and how resources are allocated, and they can also be leveraged to promote change [69, 136, 137]. In sociopolitical history, totalitarian regime norms can significantly restrain the deinstitutionalisation process [121] or even lead to the genocide of people with psychiatric diagnoses [138]. According to our review findings, a more inclusive, fair, and democratic society will have the opposite effect and facilitate the deinstitutionalisation movement [44]. In this scoping review, it was also possible to identify that the exclusiveness of the medical model is one of the causes of the lack of significant progress in mental health deinstitutionalisation. The mental health system’s dependence on a biomedical model has resulted in coercion and impediments to a diverse life in the community [139]. Only focusing on treatment and symptom reduction has led to the need for a radical change of orientation to a model that profoundly impacts the human rights of people with mental illness [140, 141]. Moving away from uniquely medical, disease, and institutional thinking suggests a direction toward a model that focuses on recovery, social inclusion, human rights, and full citizenship as the needed guiding values of change [17, 141]. Our results suggest a shift from a hospital model to a community-integrated service model [118]. Recent research accompanies this direction by introducing an integrated recovery-oriented model for mental health services capable of reinstating hope, regaining competencies, and reconnecting users with the community [142]. In addition, a high-quality community-based integrated care should be defined by the protection of human rights, supporting recovery, making use of evidence-based and users’ goals, development of community networks, and peer support expertise in service delivery as the model guiding principles [143]. Furthermore, often in mental health literature, social discrimination and stigma are seen as obstacles to deinstitutionalisation and community inclusion [74, 75, 144]. Not as common, but still prevalent, is the result of diminishing social discrimination through suitable housing and social interaction with other society members, which can only be achieved by being included in society [145, 146]. These opportunities for people diagnosed with mental illness to access valued social roles and to be part of mainstream society result in the improvement of mental health, wellness, and general health [62]. The exercise of citizens’ rights and responsibilities is a necessary precondition for recovery rather than a reward determined by overcoming mental health symptoms [147]. A citizenship approach means the promotion of participation and inclusion into a meaningful society for people with psychiatric disabilities, as well as access to full rights and responsibilities as enjoyed by any other member of a democratic society [148, 149]. A shift from the exclusiveness of the medical model to a community inclusion model has the potential to leverage the needed change of the norms in the mental health system and contribute to significant advancements in deinstitutionalisation.

The availability of system resources is influenced by norms and can dictate the capacity of a system to fulfil its purpose [69]. In our case, the lack of community services and a community mental health workforce constitutes an obstruction to the advancement of deinstitutionalisation. Our findings suggest that the impediment of deinstitutionalisation progress does not result exclusively from the lack of resources but also from the quality and adequacy of the community alternatives made available to people with psychiatric disability needs, as argued before [42]. The development of adequately tailored to the needs and accessible community resources has been one of the most significant problems of mental health deinstitutionalisation [39, 43, 140]. Some authors consider that the high imprisonment rate is not a direct consequence of the deinstitutionalisation process but rather a consequence of this underinvestment in community services that promote social inclusion as psychiatric hospitals close [150]. Aligned with our findings, research recommendations yield effective evidence-based interventions and contextualised community-driven strategies, emphasising the development of community support networks [151, 152]. A relevant example in scoped papers of community alternatives and individualised context-oriented support services is evidence-based programmes that promote access to income and work in the mainstream community [59, 80, 87, 90, 91, 94, 108, 110, 113]. These supported employment, or individual placement and support programmes, break down barriers to facilitate access to regular contexts and are based on the personal preferences and choices of people with mental illness rather than services guided by providers’ judgement regarding deserving or competence conditions [152, 153]. Previous studies have found that access to mainstream community resources, like competitive employment, improves mental health symptom control, quality of life, empowerment, and community inclusion [154–157]. According to our findings, segregated contexts, such as places of psychiatric rehabilitation or institutionalisation, restrict and limit the social relationships of people who experience mental illness [86, 115]. Then again, transitioning to independent living without support for integration and community involvement does not necessarily increase community participation or a sense of inclusion [158]. In addition to being individualised, person-centred, and tailored to people’s needs in the community, context-oriented support is also focused on developing natural support from the community context; hence, a social support network is key to community tenure after discharge [92, 94, 108]. For a successful deinstitutionalisation process focusing on social resources, it is crucial to expand the investment in meaningful social connections and community support networks; it contributes towards the improvement of mental health symptomatology, enhances recovery, and is considered an essential indicators of community inclusion [144, 159–162].

Concerning housing resources, the empirical results of included studies recommend that deinstitutionalisation processes should target non-institutional settings to prevent the transinstitutionalisation of people who transition to the community [38, 89, 103, 106, 120, 123]. Research has demonstrated that the transition to community care enhances the quality of life and improves social networks, even for long-stay patients discharged from psychiatric hospitals [163, 164]. Nevertheless, the possibility of being “institutionalised” in the community is real [6, 145], as Dear [165] highlighted, regarding the tendency to congregate people discharged from psychiatric hospitals in geographically limited areas in inner cities, what he called “psychiatric patients’ ghettos”. Additionally, Metraux et al. [166] reinforced that being residentially segregated hinders physical, social, and psychological community integration. The process of transference and re-institutionalisation of people from psychiatric hospitals to another institutional context, such as group homes or nursing homes, even if located in the community, is what Talbott [49] designated as transinstitutionalisation. These contexts were created to replace psychiatric hospitals but have demonstrated not to be better solutions than the previous institutions they were supposed to replace [29]. The deinstitutionalisation process can be one of the factors that lead to transinstitutionalisation [167] but has the potential to succeed if the transition from institutions occurs to home-based contexts with quality support [29]. The smaller the accommodation, the more individualised and home-like, and the greater the privacy, control, and power of choice, potentiates positive results and satisfaction for people with psychiatric disabilities [168, 169]. Our findings are aligned with the premise that independent housing, with support, in the mainstream community is an ideal solution that best advances community inclusion [170, 171]. A job and a home, as mentioned by Pilisuk [113], allow people with psychiatric disability to live independently outside psychiatric institutions and fulfil their human rights. However, some people transitioning from institutionalisation may require long-term support, and other solutions can also be considered to facilitate their independence, such as supported housing, as long as their on-site support services promote human rights (respecting dignity, privacy and legal rights) and are recovery-oriented (collaboration between staff and users, support user’s control of services and independent living skills, and has a culture of hope in users’ progress) [172].

Finally, as to economic resources, our findings show that the political decision to continue financing psychiatric institutions instead of reallocation funds to the community demonstrates that the political priority is still not focused on the social reintegration of people who have undergone psychiatric institutionalisation as an outcome [52, 82, 87, 101, 102, 125]. Instead, the people with this experience who are economically vulnerable and still aim to live and work in the community will persistently be unable to access resources due to the lack of service availability [20]. Both public and private financing models favour repeated in-ward treatment services, hinder the allocation of resources to community mental health, thus postponing deinstitutionalisation [38, 52, 107]. Moreover, the private mental health sector does not usually have direct orientation from regulatory agencies, leading to longer psychiatric stays, which results in increased profits [173, 174]. Some studies indicate that deinstitutionalisation and moving mental health services to the community is cost-effective [38, 84, 127], yet other studies advance the argument that a fully implemented community-based-system that meets people’s needs may prove to be at least as expensive as the inpatient system; nevertheless, cost-effective studies need to address the advantages of community inclusion as a natural support system [175].

The government uses policies to regulate the system and ensure that practices and procedures are aligned with the proposed changes. The concrete fact of developing a policy is not a precondition for system stakeholders to effectively implement the proposed changes [69]. The absence of deinstitutionalisation policies and conflicting documents reinforcing institutionalisation, identified in this scoping review [79, 87–89, 107], indicate the need for legal and policy reform to favour a successful deinstitutionalisation process. Mahdanian et al. [141] argue that in most countries, mental health laws strengthen discrimination, disempowerment, and social exclusion. Nevertheless, it was possible to identify some favourable policies that regulate psychiatric commitment and promote independent living [44, 91, 104]. Considering the consistent incongruence between the vision and principles advocated in policy documents [176], several reiterated political, legal, and social actions are required to invert the dominant institutional paradigm into policies that directly influence practices, promoting recovery, citizenship, and rights of people with psychiatric disabilities [17, 177].

For a system to carry out its mission, multiple operations are necessary, including information communication, resource mobilisation, decision-making processes, and clarifying the power sources. Foster-Fishman et al. [69] argue that focusing attention on power can be an opportunity to address the root causes of the problem, as certain decision-making models can hinder individual, collective, and community development. In psychiatric asylums, people were controlled through other coercion mechanisms (e.g., restraints, excessive medication, electric shocks, and lobotomy), influencing practices and professionals to become convinced that people with mental illness had to be treated by force and coercion [178]. The policies that allowed these premises perpetuated this abuse and were usually centred on professionals’ expertise [179]. The exclusiveness of the medical model norms has been extending power asymmetries and systems of social control in all mental health settings, even in the community [180]. In this scoping review, it was possible to identify that professional control, paternalistic, custodial, and discriminatory attitudes, restraining personal freedoms, autonomy, participation, and social integration, hindering deinstitutionalisation [47, 52, 65, 77, 78, 94, 106, 116, 119, 121]. These models and professional attitudes should continue to be challenged by universal instruments, such as the Convention on the Rights of Persons with Disabilities (CRPD), the NICE Guidelines on Rehabilitation for adults with complex psychosis [181], recommending transition process considering the sharing of information, a highly integrated care, and recovery focussed, the findings of the World Psychiatry Association on Implementation of Alternatives to Coercion [182] or self-representation movements [180, 183]. Galbert et al. [184] argue that the system’s power and control are not related to factors associated with mental illness experiences but are influenced by the control and coercion practices related to psychiatric institutions’ daily routines. This legacy has influenced mental health professionals’ portrayed pessimism regarding recovery [185], largely included in political discourse with no meaningful connection with the promotion of social justice, balancing power dynamics, increasing the risk of transforming recovery, and consumers’ participation in decision-making in a mere abstraction [186]. Although consumer participation is perceived as a good policy [187], there is limited support for decision-making among people with psychiatric disability. Instead, family members and service providers retain power, through guardianship, to decide the main aspects of people’s lives [63]. In turn, restraining people’s choices inhibits the potential for social inclusion [188]. In our review findings, consumer participation contributes to regaining control over one’s life, expanding social networks and community living. It can incorporate different forms that could lever deinstitutionalisation, be they the employment of consumers, including the consumer perspective into service development and discharge planning, or peer-support programmes [59, 78, 85, 86, 103, 119]. The importance of peer support and consumer participation is also evident in other similar literature reviews, specifically concerning consumer choice and autonomy, participation in support groups, and involvement of consumers in decision-making at all levels [74, 75, 141]. Consumer participation and peer support are intimately connected to advocacy movements because it is through peer support that people can grow from passive victims of discrimination into survivors and advocates [131]. Although it was noted in our scoping review that deinstitutionalisation is hindered by the lack and the restraining of advocacy groups’ efforts [87, 125], it was also identified that consumer advocacy and civil rights initiatives could expand community resources and lessen social discrimination, facilitating the access to the community for those transitioning from psychiatric institutions [65, 81, 107]. The “ex-patients’ movement” is an example of these drivers, creating new norms and questioning the medical model, developing consumer-controlled services, like mutual help groups, and advocating for people diagnosed with mental illness to speak for themselves [189]. The institutionalised mental health system’s entrenched functioning has proven to be difficult to change, and alternative consumer participation has demonstrated the potential to transform the way services are delivered [186].

## Limitations

Some limitations are important to recognise in this study. First, although our search strategy was carefully planned, including the supervision of two specialised librarians, and sources were rigorously screened, we cannot guarantee that all relevant sources were obtained. Certainly, a broader search strategy, resorting to other databases from different fields of expertise, could have amplified the number of studies on mental health deinstitutionalisation. Second, focusing on papers written in English may have caused the loss of some important resources, such as empirical studies published in non-English languages. Third, although we consider it a strength only include peer-reviewed empirical studies, excluding studies that were not empirical, like grey literature or guidelines could mean the loss of valuable information about theories, programmes, and implementation data that could be pertinent to clarify factors associated with deinstitutionalisation experiences, but are not under peer revision. Fourth, we only included studies concerning the deinstitutionalisation of adults diagnosed with mental illness; therefore, studies with study groups or samples on other disability groups and social movements were not included, and could also be a source of learning about an effective deinstitutionalisation process. Fifth, this review lacks some geographic diversity, as a wide range of studies were conducted in Western and high-income countries, which are considered pioneers in the development of deinstitutionalisation policies; nevertheless, a more worldly perspective could have been attained by not limiting included studies to the English language. Sixth, studies concerning family caregivers and deinstitutionalisation hindrances and drivers, and studies focusing on peer support, consumer-run organisations, and consumer-led roles in the mental health deinstitutionalisation process are still a minority, as concluded in a similar scoping review [67], the existence of more studies these topics could have led to different categories. Seventh, a scoping review does not need to adopt a quality assessment of the included studies. This can be a limitation. Nevertheless, according to the SCImago journal ranking of the 57 included studies, 58% (*n* = 33) are in quartile 1, 32% (*n* = 18) in quartile 2, and 10% (*n* = 6) are in quartile 3, demonstrating that the majority of included studies may be considered as high quality. Eighth, descriptive synthesis and the organisation of categories into the system change framework [61] involve some degree of subjectivity and interpretation. Ninth, conclusions can be difficult to illustrate due to the variety of methods among the included studies. To counter these potential biases, all the coding was performed independently by two or three reviewers and discussed with the entire research team. Finally, we used the definition of deinstitutionalisation, according to the Guidelines on Deinstitutionalisation, Including in Emergencies, recently published by the United Nations Committee on the Rights of Persons with Disabilities [1], to ensure that our study was in line with consumer survivor’s movement and human rights, which in turn, may have limited the inclusion of other approaches.

## Implications for future research

Future research should adopt a community-based and rights-oriented public health perspective to address the deep-rooted barriers hindering effective deinstitutionalisation. This includes challenging the dominance of the medical model, institutional thinking, and professional hierarchies through participatory, community-driven approaches. Research should prioritize the voices of people with lived experience, focus on evidence-based, explore how communities can serve as not just sites of care but active agents of inclusion and systemic change, context-sensitive models of support, and examine how policies, funding, and professional practices can be restructured to promote inclusion, dignity, and human rights. Further exploration of consumer advocacy and grassroots movements as drivers of systemic change is also crucial. Also, how intersectional barriers (e.g., culture, geographical location, gender, class) shape access to care, quality of life, and community inclusion post-deinstitutionalisation. Ultimately, future research must not only critique existing systems but also actively contribute to building equitable, inclusive, and community-rooted mental health alternatives.

## Implications for practice and policymaking

The findings of this scoping review provide valuable information regarding hindrances and drivers of the deinstitutionalisation process of adults diagnosed with mental illness to be considered by mental health practitioners and policymakers. Policymakers can resort to these findings to develop policies and allocate funds capable of deterring segregation and social exclusion, isolating people with disabilities in psychiatric institutions. Our findings identified the importance of policies to recognise the full and diverse life of people diagnosed with mental illness and policies that incorporate and prioritise people’s social needs (housing, work) and community inclusion. Policymakers can also use these insights to ensure the allocation of funds to community services that meet people’s needs based on systematised evidence. Another finding is the importance of incorporating the perspective and effective participation of people diagnosed with mental illness in the definition of policies and the leadership of the deinstitutionalisation process. Policymakers may use this information to contribute to a change in power dynamics and reduce social discrimination. Mental health service providers, practitioners, and trainees may find the present study relevant for informing and contributing to the training of future professionals. The current study contributes to an in-depth reflection on the relevance of transforming paternalistic, controlling, and hierarchical roles into facilitators of community inclusion based on people’s choices. Our review findings emphasise the importance of the transition from psychiatric institutional settings into mainstream community resources. Service providers can use this study to recognise which discharge accommodation and type of support are effective in promoting community tenure, social networks, and consumer control over one’s life, well-being, and mental health. By focusing on the identified hindrances and drivers of mental health deinstitutionalisation, service providers and policymakers can contribute to the mental health system change by ensuring that the remaining people in psychiatric institutions have opportunities for social integration and inclusion, fulfilling their human rights.

## Conclusions

This scoping review adds to the existing scientific knowledge concerning the deinstitutionalisation process in the mental health field. The mapped hindrances and drivers can serve as an important basis to inform the ongoing or future deinstitutionalisation processes of adults diagnosed with mental illness. It can also contribute to the clarification of the paradoxical dimension of deinstitutionalization and to further understanding of why, after over a half-century, the deinstitutionalisation movement has not been able to ensure community inclusion. Some system elements persist as inconsistent with this goal, as the exclusiveness of the medical model and institutional thinking still over other approaches, leading to opposite system norms and values of community inclusion. The hegemony of these system norms has negatively influenced the other interdependent system elements. Thus, policies, allocation of funds, services, practices, professional attitudes, and power dynamics act as hindrances to the deinstitutionalisation movement. Some barriers encountered may be reversible by facilitating an effective allocation of financial resources and through the outline of policies and laws based on human rights and social justice that include a clear vision of how to overcome barriers to basic needs (e.g., housing, employment) and how these goals are achieved. Additionally, it is necessary to transition to evidence-based programmes that facilitate community inclusion and provide a continuum of community context-based support rather than perpetuating the discharge of people with mental illnesses into segregated spaces. Some hindrances will be more difficult to reverse, such as the predominance of the medical model, professional hierarchical power, and persistent institutional isolation, requiring change in deeply rooted structures. Promoting consumer participation and advocacy movements has demonstrated having the potential to act as critical levers of change, challenging the entrenched culture in the model of care and practices. Our results reinforce the need for a transformative change in mental health, specifically identifying how the mental health system elements can function as possible drivers of change, ensuring an effective deinstitutionalisation process and contributing to the social justice of people diagnosed with mental illness.

## Supplementary Information

Supplementary Material 1.

Supplementary Material 2.

## Data Availability

The datasets used and/or analysed during the current study are available from the corresponding author upon reasonable request.
