# Supplementary material for: Putting mental health deinstitutionalisation back on track: a scoping review of what empirically hinders and drives deinstitutionalisation of adults who experience mental illness
Source: BMC Public Health. 2025 Nov 26;25:4152. doi: 10.1186/s12889-025-24496-0 (PMC12659268; doi:10.1186/s12889-025-24496-0)
Supplement: Supplementary file 1 — Supplementary Material 1. [file 12889_2025_24496_MOESM1_ESM.docx]

**Additional Table 1** List of extracted hinders

| **System elements** | **Categories** | **Identified Hinders** | **Ref.** |
| --- | --- | --- | --- |
| Norms | The exclusiveness of the medical model | the old traditional institutional culture and psychiatric view as a barrier to change | 78 |
|  |  | Staff attitudes moved by the medical model, of custodial and paternalistic characteristics of acute settings exclude consumer focus | 78 |
|  |  | Rehabilitation centers should foster reintegration into society, but instead overmedicate people and people stay there until they die | 83 |
|  |  | A model that is only centered on causes and treatment | 83 |
|  |  | Policy instruments that maintain the prevailing logic in the Mental health field | 88 |
|  |  | Reforms that reinforce the use of institutional models as a social policy response | 89 |
|  |  | Dominance of the medical model that has different values than social approaches | 97 |
|  |  | Professional reliance on an antiquated/ narrow model of mental disorder | 52 |
|  |  | The current care system still functioning as the old hospital | 103 |
|  |  | The lack of translation of the person-centered, community-based, and well-being focus vision into meaningful descriptors and measurements of outcomes | 105 |
|  |  | Legacy of Psychiatric Hospitals Policy prohibited the widespread mental health reform | 107 |
|  |  | Entrenched hospital-based focus on mental health treatment | 107 |
|  |  | Hospital-based psychiatric rehabilitation programs | 115 |
|  |  | Significant reliance on inpatient psychiatric units to provide psychiatric beds | 117 |
|  |  | Specialized and Hospital services maintained centralized service under the umbrella of user protection | 118 |
|  |  | The fierce opposition from professionals following the biomedical model (psychiatrists and nurses) | 47 |
|  | Social discrimination | Discrimination and prejudice of others undermine efforts to live and recover in the community | 110 |
|  |  | Society stigma, not accepting mental illness just like any other difference | 114 |
|  |  | Discrimination promoted by the media | 121 |
| Resources | Insufficient community service | Lack of human resources for mental health that meet people’s needs | 83 |
|  |  | Lack of quality resources, as alternatives to institutionalization | 83 |
|  |  | Shortcomings of community care following deinstitutionalization | 86 |
|  |  | Shortcomings of community mental health contributed to more investment in hospital beds and new forms of institutional care (protected housing, forensic services) | 38 |
|  |  | Sub-optimal environmental and social circumstances to maintain community tenure | 90 |
|  |  | Lack of support from outreach teams | 92 |
|  |  | The lack of civil society resources encourages families to institutionalize | 52 |
|  |  | Limited opportunities to access services that facilitate independent living in the community | 99 |
|  |  | The lack of housing choices for people leads to integration in facilities away from family and friends or reinstitutionalisation | 99 |
|  |  | An underfunded Community care means little support when moving to the community | 103 |
|  |  | Unavailability of suitable accommodation option | 106 |
|  |  | Lack of housing choices | 106 |
|  |  | Lack of supported living services | 106 |
|  |  | People have little choice where and with whom they live | 106 |
|  |  | Lack of community services following discharge | 110 |
|  |  | The lack of alternative support structures and limited community support | 112 |
|  |  | Inadequate number of community mental health professionals | 117 |
|  |  | Insufficient availability of service vacancies | 118 |
|  | Trans-institutionalisation | Institutional control of group homes and therapeutic residences, hinders a person’s identity, freedom, autonomy, and social integration | 77 |
|  |  | Rehabilitation centers should foster reintegration into society, but instead overmedicate people and people stay there until they die | 83 |
|  |  | Residential rehabilitation/ community institutional care has negative effects, such as social isolation, loss of social support, marginalization | 86 |
|  |  | Shortcomings of community mental health contributed to more investment in hospital beds and new forms of institutional care (protected housing, forensic services) | 38 |
|  |  | Healthcare systems continue to invest in an outdated segregated program | 87 |
|  |  | Boarding housing (Group homes) is transinstitutionalization, not human rights, and not an appropriate housing mode | 89 |
|  |  | Warehousing” people still are legitimized by social policy responses | 89 |
|  |  | Some residential facilities recreate small-scale institutional structures and processes | 97 |
|  |  | The lack of housing choices for people leads to integration in facilities away from family and friends or reinstitutionalization | 99 |
|  |  | Privatization of the system leads to transinstitutionalization | 103 |
|  |  | Community accommodations that control, dominate, and restrict autonomy and choice | 106 |
|  |  | Inadequate planning for independent living while in the hospital | 110 |
|  |  | Hospital-based psychiatric rehabilitation programs | 115 |
|  |  | Transinstitutionalization is a major component of institutionalization in the transition to community process | 120 |
|  |  | The existence of a trend to trans and re-institutionalization | 47 |
|  |  | Institutionalism is transferred to community services, through the organization's culture | 123 |
|  |  | Transinstitutionalization by the maintenance of the staff and user roles | 123 |
|  |  | Halfway houses | 123 |
|  |  | Re-institutionalization into the new form of control institutions | 124 |
|  | Lack of support for community inclusion | Lack of financial resources for mental health that meet people’s needs | 83 |
|  |  | Lack of human resources for mental health that meet people’s needs | 83 |
|  |  | Policy agendas not focusing on evidence-based social support (supported housing and employment) | 87 |
|  |  | Community placement is not community integration, since most people have not yet found a home in the community | 59 |
|  |  | Need for services that strengthen integration in social resources (employment, Housing support) | 90 |
|  |  | Integrated in an environment that doesn’t promote living a meaningful life | 90 |
|  |  | Lack of support targeted at meaningful activities, at becoming involved with others, and for a structural daily activities’ routine | 92 |
|  |  | Support for social inclusion is a low priority for staff | 97 |
|  |  | Competition and poor communication between housing and community support actors impede meaningful social inclusion | 97 |
|  |  | The lack of translation of the person-centered, community-based, and well-being focus vision into meaningful descriptors and measurements of outcomes | 105 |
|  |  | Separated services of provision of a house and living support | 106 |
|  |  | Poor employment support (job training in community centers without coordination with employers) | 110 |
|  |  | Poor continuity of care | 110 |
|  |  | Family lack of knowledge and support about recovery | 110 |
|  |  | Lack of support for meaningful occupation and enhancement of opportunities following transition | 111 |
|  |  | Lack of support for social connectedness | 111 |
|  |  | The lack of alternative support structures and limited community support | 112 |
|  | Most funds allocated to institutionalisation | The lack of funding for mental health hinders MHD | 79 |
|  |  | Most funds are allocated to inpatient care | 82 |
|  |  | A low proportion of the health budget is spent on mental health | 82 |
|  |  | Lack of financial resources for mental health that meet people’s needs | 83 |
|  |  | Healthcare systems continue to invest in an outdated segregated program | 87 |
|  |  | Psychiatric Hospitals absorb a high proportion of the budget | 52 |
|  |  | The government allocates budget funds due to historical expenditures and available infrastructures rather than population need | 52 |
|  |  | Lack of funding for residential care, most of the available budget is for mental hospitals | 101 |
|  |  | The majority of financial (60%) and resources (2/3) are spent in Psychiatric Hospitals, resources are not distributed efficiently | 102 |
|  |  | An underfunded Community care means little support when moving to the community | 103 |
|  |  | Few resources are allocated to community-based services than inpatient services | 120 |
|  |  | Lack of political will to allocate funds to community care | 125 |
|  |  | Not investing in community services with the disinvestment | 125 |
|  |  | Discrepancies in community mental health needs and available funding | 125 |
|  | Economic incentives for institutionalisation | Privatization of health care leads to economic interest in institutional care | 38 |
|  |  | Perverse incentives to maintain high bed occupancy of long-stay institutions | 52 |
|  |  | A high percentage of health insurance reimbursement is for Psychiatric Hospitals | 101 |
|  |  | Privatization of the system leads to transinstitutionalization | 103 |
|  |  | The government's national health insurance covers hospital services only and not community treatment | 107 |
|  |  | Financial incentives prioritise inpatient treatment | 110 |
|  |  | Psychiatric expenditures are related to the number of inpatient days | 120 |
| Regulations | Institutional policies | Lack of policies that promote deinstitutionalisation | 79 |
|  |  | Lack of policy reforms that promote programs that facilitate community integration and recovery | 87 |
|  |  | Policy agendas not focusing on evidence-based social support (supported housing and employment) | 87 |
|  |  | Policy instruments that maintain the prevailing logic in the Mental health field | 88 |
|  |  | Warehousing” people still are legitimized by social policy responses | 89 |
|  |  | Reforms that reinforce the use of institutional models as a social policy response | 89 |
|  |  | Mental health policies have a lower priority in policymaking than physical illness | 107 |
|  |  | The privileged position of psychiatrist in mental health policy | 107 |
|  | Inefficient governance | Not having intersectoral collaboration in polices hinders deinstitutionalization. | 79 |
|  |  | Not having an integrated care | 83 |
|  |  | Lack of a common health care framework for mental health providers | 83 |
|  |  | Lack of intersectoral collaboration between policies | 52 |
|  |  | Commissioning is not an approach that leads to a mental health care change | 105 |
|  |  | Lack of coordination between psychiatrists and other services providers | 110 |
|  |  | Unclear roles and lack of trust between mental health services and primary health care centers | 118 |
|  |  | Divergence in priority policy goals within stakeholders’ coalitions | 122 |
|  |  | Lack of regional authority mandates to promote deinstitutionalization | 125 |
| Operations | Professional control | Institutional control of group homes and therapeutic residences, hinders a person’s identity, freedom, autonomy, and social integration. | 77 |
|  |  | Staff attitudes moved by the medical model, of custodial and paternalistic characteristics of acute settings exclude consumer focus. | 78 |
|  |  | Paternalistic and stigmatization attitudes from professional care providers | 94 |
|  |  | Professional pessimism over recovery | 52 |
|  |  | Professional hierarchical approach to clinical decision-making | 52 |
|  |  | Community accommodations that control, dominate, and restrict autonomy and choice | 106 |
|  |  | The privileged position of psychiatrist in mental health policy | 107 |
|  |  | The exclusion of family members in the partnership caregivers-medical team | 116 |
|  |  | Specialized and Hospital services maintained centralized service under the umbrella of user protection | 118 |
|  |  | A paternalistic approach from staff hinders the power to participate and discuss one’s discharge plan meaningfully | 119 |
|  |  | Professionals’ resistance to deinstitutionalization | 121 |
|  |  | Staff paternalistic and control attitudes towards people with mental health problems | 121 |
|  |  | The fierce opposition from professionals following the biomedical model (psychiatrists and nurses) | 47 |
|  |  | Professionals’ resistance to deinstitutionalization | 65 |
|  |  | Transinstitutionalization by the maintenance of the staff and user roles | 123 |
|  |  | Re-institutionalization into the new form of control institutions | 124 |
|  | Limited Advocacy | Lack of advocacy groups representing people with mental illness | 79 |
|  |  | Efforts of the groups seeking to influence policy are constrained | 125 |
