# Supplementary material for: Putting mental health deinstitutionalisation back on track: a scoping review of what empirically hinders and drives deinstitutionalisation of adults who experience mental illness
Source: BMC Public Health. 2025 Nov 26;25:4152. doi: 10.1186/s12889-025-24496-0 (PMC12659268; doi:10.1186/s12889-025-24496-0)
Supplement: Supplementary file 2 — Supplementary Material 2. [file 12889_2025_24496_MOESM2_ESM.docx]

**Additional Table 2** List of extracted drivers

| **System elements** | **Categories** | **Extracted Drivers** | **Ref.** |
| --- | --- | --- | --- |
| Norms | Model for community inclusion | A new model of mental health care, a citizenship model, with the social needs ahead (living, working, and environmental conditions) | 80 |
|  |  | Supporting people integrate into a familiar natural community context | 59 |
|  |  | Involvement in social rather than rehabilitation activities | 95 |
|  |  | Mental health shift to focus on social-material opportunities in the community | 96 |
|  |  | Housing support services shifted from institutional to community-based, from treatment-based to independent living | 99 |
|  |  | Discharge model that supports the building of relationships and citizenship (empl, edu, community activism, peer support) | 108 |
|  |  | Community integration and social support predict recovery after discharge | 108 |
|  |  | Transition solutions in mainstream society that assure people freedom, choice, and control over one’s lives | 109 |
|  |  | A shift from a hospital model of intervention to a community-integrated service model | 118 |
|  |  | Mental health care that values autonomy and social participation | 118 |
|  | Inclusive society | The greater the country’s ethnic diversity, the more accelerated deinstitutionalisation | 44 |
|  |  | The higher the Human Development Index the more advanced deinstitutionalisation is | 44 |
|  |  | The greater the Index of Democratization the greater the deinstitutionalisation | 44 |
|  |  | High Secular-Rational culture drives deinstitutionalisation | 44 |
| Resources | Resourcing community alternatives | Alternatives that respond to consumers’ needs and preferences, like a home in the community | 59 |
|  |  | Housing and community start-up cost Policies help find a house at discharge and prevent homelessness | 91 |
|  |  | Housing First program (Increases house tenure, reduces cost and psychiatric hospitalisation) | 93 |
|  |  | Having a significant something (Work and support, matching work with people’s needs and desires). | 94 |
|  |  | Transition to places that enable living, that allow people to move, act, and be alive in everyday life | 96 |
|  |  | Bring people closer to their local communities | 97 |
|  |  | Strong community mental health care | 44 |
|  |  | Provision of community-based discharge planning services | 98 |
|  |  | Infusion of funds from closing state hospitals into the community system | 100 |
|  |  | Public sector expenditures allocated to residential care | 101 |
|  |  | Properly planned and resourced deinstitutionalisation that ensures quality of care with enduring solutions | 109 |
|  |  | Programs focus on the positive strengths of users | 114 |
|  |  | Support care that meets people’s needs | 118 |
|  |  | Increasing funding and services of the community psychosocial service network | 118 |
|  |  | Considerations of patients' full and diverse life | 119 |
|  |  | Non-governmental organisation service providers | 123 |
|  |  | Deinstitutionalisation to a non-institutional setting | 123 |
|  |  | Individualised planning and tailored integrated care, in partnership with community services, promote a successful community transition | 126 |
|  |  | Supports and services tailored to people’s needs | 126 |
|  | Independent housing | Living by oneself / alone (Promotes autonomy and social integration) | 77 |
|  |  | Alternatives that respond to consumers’ needs and preferences, like a home in the community | 59 |
|  |  | Immediate support for income and obtaining a house upon discharge | 91 |
|  |  | Support that gives direct access to the house, and no need to be house-ready | 93 |
|  |  | The smaller the accommodation (2 or 3 people), the more favourable | 95 |
|  |  | Housing support services shifted from institutional to community-based, from treatment-based to independent living | 99 |
|  |  | A law that promotes the return to the community and independent living | 104 |
|  |  | An experimental independent living program with support enhances the capacity to live in the community after discharge (more likely to be employed, strengthened networks, larger informal support network) | 113 |
|  | Individualised context-oriented support | The importance and need for long-term supportive care | 86 |
|  |  | Social networks create stability and community residence | 86 |
|  |  | Support people to build connections, a sense of belonging, and a positive identity | 59 |
|  |  | More emphasis on individual support for normalising social and recreational activities, supported independent living, supported employment, and supported education | 59 |
|  |  | Family member support is fundamental after discharge | 92 |
|  |  | Support that gives direct access to a house and no need to be house-ready | 93 |
|  |  | Having a significant something (Work and support, matching work with people’s needs and desires). | 94 |
|  |  | Mental health system support with adequate community support and continuity of care | 95 |
|  |  | Staff and family support were identified as significant for transitioning to community living | 97 |
|  |  | The availability of intensive case management services prevents institutionalisation | 100 |
|  |  | Community integration and social support predict recovery after discharge | 108 |
|  |  | Support from family members at transition and post-transitioning in the community | 111 |
|  |  | Detailed planning, personalised care, and a gradual transition | 111 |
|  |  | Programs that build bridges between psychiatry, organisations, and individuals in the community | 114 |
|  |  | Continued support after discharge from the hospital, teams, and community agencies | 115 |
|  |  | Support that focuses on the development of personal support networks | 115 |
|  |  | Family member support | 118 |
|  |  | Collaborative practice in developing a care plan | 119 |
|  |  | Partnership between the government, professionals, and experts | 65 |
|  |  | Supports and services tailored to people's needs | 126 |
|  | Economic pressures | In periods of high inflation, psychiatric beds decrease significantly, driving mental health deinstitutionalisation | 84 |
|  |  | Deinstitutionalisation is driven by being seen as a cost-reduction strategy Vs the high cost of inpatient care | 38 |
|  |  | Housing First program (Increases house tenure, reduces cost and psychiatric hospitalisation) | 93 |
|  |  | Transition to community care is cost-effective | 127 |
| Regulations | Policy and legal reform | Housing and community start-up cost Policies help find a house at discharge and prevent homelessness | 91 |
|  |  | Deinstitutionalisation is driven by the unacceptable practices in old asylums (Moral principles) | 38 |
|  |  | Having a formal law governing psychiatric commitment is a promoter of deinstitutionalisation | 44 |
|  |  | A shift in community-based support services is driven by modifications in policies and laws | 99 |
|  |  | A law that promotes the return to the community and independent living | 104 |
|  |  | Having a mental health law | 47 |
|  |  | Closing institutions | 100 |
|  |  | Exogenous shocks (war, natural disasters, infringement of human rights) cause a psychiatric beds to decrease | 47 |
|  |  | The poor state of psychiatric hospitals promoted the urgency for change | 65 |
|  |  | Cooperation from all levels of government | 65 |
|  |  | Utility of research in furthering deinstitutionalisation | 65 |
| Operations | Consumer participation in services | Consumer participation in services | 78 |
|  |  | Employment of consumer consultants | 78 |
|  |  | Peer support base program (Expand social networks, community living) | 85 |
|  |  | Incorporating the consumer perspective into service development initiatives | 86 |
|  |  | Consumer control over daily life | 59 |
|  |  | Ensure people`s choice and control when moving to community living | 97 |
|  |  | Engaging in alternative critical approaches from the experience of psychiatric survivors | 103 |
|  |  | Patient participation/ empowerment in discharge | 119 |
|  |  | Sharing all information with the patient for patient decision-making | 119 |
|  |  | Participating in user-led mental health organisations | 119 |
|  | Consumer Advocacy | Consumer advocacy and civil rights initiatives expand community resources | 81 |
|  |  | Shift of society's ideas (lessening stigma) from public figures speaking out about their personal mental illness experiences | 107 |
|  |  | NGOs and consumer advocacy | 65 |
